# Olive Oil Quality and Authenticity Assessment Aspects Employing FIA-MRMS and LC-Orbitrap MS Metabolomic Approaches

**DOI:** 10.3389/fpubh.2020.558226

**Published:** 2020-09-25

**Authors:** Theodora Nikou, Matthias Witt, Panagiotis Stathopoulos, Aiko Barsch, Maria Halabalaki

**Affiliations:** ^1^Department of Pharmacognosy and Natural Products Chemistry, Faculty of Pharmacy, National and Kapodistrian University of Athens, Athens, Greece; ^2^Bruker Daltonik GmbH, Bremen, Germany

**Keywords:** olive oil, EVOO, FIA-MRMS, LC-Orbitrap MS, polyphenols, quality control, authenticity

## Abstract

Edible vegetable oils comprise integral components of humans' daily diet during the lifetime. Therefore, they constitute a central part of dietary-exposome, which among other factors regulates human health. In particular, the regular consumption of olive oil (OO) has been largely accepted as a healthy dietary pattern. Responsible for its recognition as a superior edible oil is its exceptional aroma and flavor. Its unique composition is characterized by high levels of monounsaturated fatty acids and the presence of minor constituents with important biological properties, such as the so-called OO polyphenols. Being a high added value product, OO suffers from extensive fraud and adulteration phenomena. However, its great chemical complexity, variability, and the plethora of parameters affecting OO composition hamper significantly the selection of the absolute criteria defining quality and authenticity, and a reliable and robust methodology is still unavailable. In the current study, Flow Injection Analysis-Magnetic Resonance Mass Spectrometry (FIA-MRMS) was investigated under a metabolic profiling concept for the analysis of Greek Extra Virgin Olive Oils (EVOO). More than 200 monovarietal (Koroneiki) EVOO samples were collected from the main Greek OO producing regions and investigated. Both intact oil and the corresponding polyphenols were analyzed in fast analysis time of 2 and 8 min, respectively. In parallel, an LC-Orbitrap MS platform was used to verify the efficiency of the method as well as a tool to increase the identification confidence of the proposed markers. Based on the results, with FIA-MRMS, comparable and improved projection and prediction models were generated in comparison to those of the more established LC-MS methodology. With FIA-MRMS more statistically significant compounds and chemical classes were identified as quality and authenticity markers, associated with specific parameters, i.e. geographical region, cultivation practice, and production procedure. Furthermore, it was possible to monitor both lipophilic and hydrophilic compounds with a single analysis. To our knowledge, this approach is among the few studies in which two FT-MS platforms combining LC and FIA methods were integrated to provide solutions to quality control aspects of OO. Moreover, both lipophilic and hydrophilic components are analyzed together, providing a holistic quality control workflow for OO.

## Introduction

Human health is determined by a combination of different factors with the most important being genetics, physiology, environment, and food. Furthermore, social, economic, and cultural factors also have significant influence on overall human health. Both internal (e.g., genetics) and external or environmental factors consist of the human exposome, which represents the totality of exposures experienced during life (1). Among these factors, food and dietary-exposome of chemicals are critical either to promote, demote, or even damage human health. Edible vegetable oils such as palm, canola, coconut, sunflower, and olive oil comprise standard ingredients of humans' daily diet and therefore own prominent impact on health during the lifetime (2).

Olive oil (OO) regular or even daily consumption has been largely accepted as a healthy dietary pattern, as demonstrated by several scientific studies highlighting its nutritional value and positive affects to human health (3). Being the main source of fat in the Mediterranean diet, a direct correlation of its protective effects against chronic ailments such as cardiovascular diseases, obesity, and cancer has been suggested. As a consequence, consumers demand is globally increasing according to International Olive Council (IOC) information, requesting a healthier way of life (4).

Being a food product of high added value for the European Union economy and mainly for the OO producing countries of the Mediterranean basin, the assurance of its quality is of outmost importance. The OO quality classifications are mainly based on free acidity, expressed as equivalents of free oleic acid, being associated with sensory and physicochemical features, which should be in accordance to assessed standards. Thus, Extra Virgin Olive Oil (EVOO), Virgin Olive Oil (VOO), ordinary virgin olive oil, virgin olive oil not fit for consumption, refined olive oil, olive oil, and olive oil pomace are the main quality categories^1^. Additional features contribute to OO quality and especially for EVOO and VOO, providing extra added value. As in other foodstuff commodities, PGI (protected geographical indication), and PDO (protected designation of origin) OO products overwhelm the global market (5). Therefore, the European Commission has adopted a framework providing guidelines for PGI and PDO, in order to enhance credibility and ensure authenticity (6).

Nevertheless, OO and especially EVOO and VOO suffer from extensive fraud and adulteration phenomena, being in the top-5 list of most fraudulent food products worldwide^2^. In most cases, the fraud consists of mixing EVOO with lower quality OOs such as deodorized oil, other vegetables oils, or even not edible oils, e.g., lamp oil. Additionally, the use of wide definitions for PDO and PGI products, and the elusive regulations concerning labeling and the employed analytical procedures for OO quality control, have led to the increase of fraud and mislabeling incidences. Regardless of intentionally or not, there were also cases where toxic or hazardous entities have been traced, such as rapeseed oil denatured by anilines, which could cause toxic effects and deaths (7). Hence, the control of OO quality and authenticity is a critical factor not only from an economical point of view but also for the protection of consumers and public health.

OO has a unique chemical composition that is responsible for its health and nutrition beneficial effects as well as its recognition as superior edible oil. Moreover, two health claims for the positive impact of OO regarding monounsaturated fatty acids (FAs) (8) and polyphenols (9, 10) have been announced by European Food Safety Authority–EFSA substantiating its health beneficial effect. More specifically, OO is generally composed of two major parts, with the third being the volatiles, which are critical for its particular aroma. The main part is the glycerol or saponifiable fraction (more than 98% of the total oil weight), which includes primarily triacylglycerides (TGs) and secondarily diglycerides (DGs) and free fatty acids (FFAs) (11). OO is rich in monounsaturated fatty acids (MUFA), especially oleic acid, which comprises almost 56–84% of the total FAs with the well-known activity in decreasing LDL-cholesterol complex and increasing of HDL-cholesterol in plasma (12).

**Figure 1:**
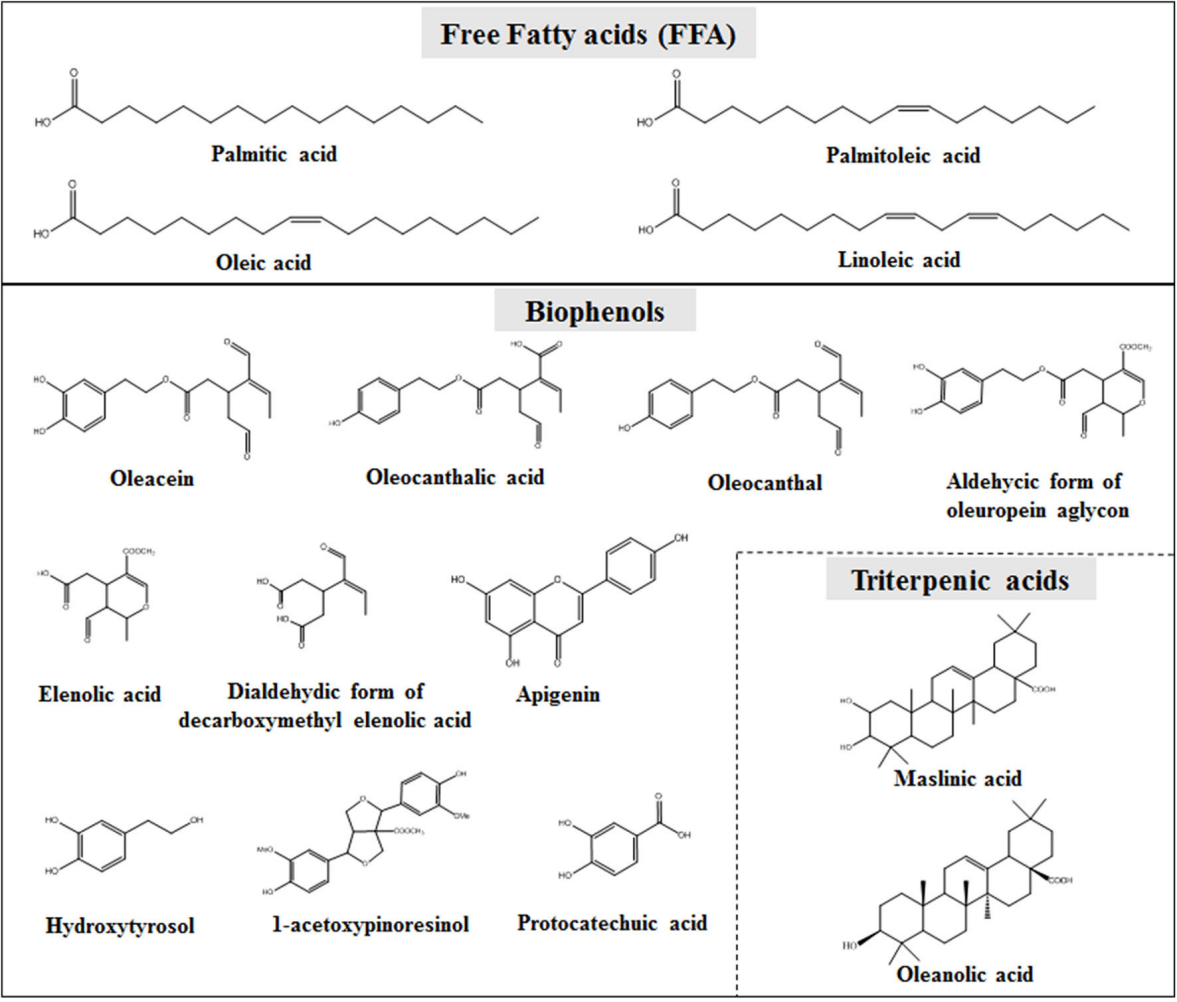
Representative compounds of OO glycerol fraction (upper part) and biophenols fraction (lower part).

The second part is the non-glycerol or unsaponifiable fraction (~1–2% of the total oil weight) (11). This fraction is characterized by high chemical complexity and most of its components found to be biologically active (13, 14). These compounds are subdivided into lipophilic and hydrophilic (15, 16). The lipophilic part contains hydrocarbons (e.g., squalene), tocopherols, sterols, and pigments such as carotenoids. On the other hand, the hydrophilic part is mainly composed by an important class of compounds, the so-called OO polyphenols, phenols, or biophenols. Numerous compounds, belonging to more than 35 different chemical classes, e.g. phenylethanoids, secoiridoids, flavonoids, lignans, as well as triterpenic acids, constitute the OO unsaponifiable fraction [Figure 1; (17)]. In the last decade much attention has been given to this small fraction of OO constituents, especially after EFSA claim regarding their protective effect of blood lipids from oxidation and the plethora of scientific evidence about the pharmacological potential of particular compounds (18).

However, OO composition is highly complex and variable depending on several factors such as genetic (e.g., tree cultivar), agronomic, and environmental (e.g., microclimate, cultivation practice, ripening stage), as well as technological parameters [e.g., collection, milling, storage; (19)]. This fact complicates considerably the OO quality and authenticity control. Adding to this inherent complexity and the fraud incidents, quality is rendered as an extremely multifaceted issue. On one side it's unique, complex, and fluctuating composition, and on the other side are the “rules” of market.

So far, to assess quality at least primarily, organoleptic properties of OO by a tasting panel of specialists, physicochemical properties (free acidity, peroxide, absorption UV K270 and K232, moisture, etc.), and presence of certain contaminants are commonly evaluated (20). However, until now, no analytical procedure has been established or accepted for detection of fraud or authenticity, and generally the verification of the information appears on the label. Up to now, the only analytical method that is suggested by IOC and generally adopted by regulation bodies is based on HPLC-UV and concerns OO polyphenols. However, this method is suffering by certain limitations, restricting its use for accurate and complete quality control purposes (21, 22). On the other hand, several analytical methods and techniques have been suggested for the quality control of OO. Based on literature data, chromatographic techniques (GC, LC, CE) coupled to several detectors (diode array detection-DAD, mass spectrometry-MS) (23, 24) are mostly employed, taking the advantage of the separation of compounds in combination with the detection characteristics and sensitivity. In other approaches, authors suggest alternative techniques to reduce or even eliminate the required chromatographic analysis time, like Fourier Transformed Infra-Red (FT-IR) spectroscopy (25) and Nuclear Magnetic Resonance (NMR) (11, 26), but with decreased sensitivity and accuracy (27, 28).

Furthermore, many studies have been carried out under metabolic profiling concept employing different analytical platforms, mainly LC-MS and NMR (29, 30). Regardless of the analytical method, usually multivariate data analysis (MDA) is applied to cope with the generated data (19). Using this approach, projection and prediction models are generated for the classification of OO samples according to the question of the study and chemical groups, or certain metabolites are in a few cases suggested (31). Additionally, the great majority of studies focus either on the saponifiable (fat) or the unsaponifiable (biophenols) part. Besides, biophenols are not usually taken under consideration when quality parameters are investigated and studies focus mainly on the glycerol part (29, 30). Other constrains exist in both LC-MS-based as well as in NMR-based metabolic profiling of fingerprinting approaches. Laborious sample preparation procedures, long analysis time, selectivity, matrix effect for example in LC-MS studies, and, e.g., low sensitivity, high cost, and identification issues in NMR-based limit their applications. Based on the above, alternative methods that provide speed, efficiency, and specificity are under investigation. Toward this direction, direct infusion MS analysis of OO has been suggested and nowadays is in the center of scientific interest for quality control purposes generally in foods (32–34).

Another issue that complicates further the analysis of OO is the accurate identification of the detected compounds and therefore possible chemical markers. Even today, the complete composition of unsaponifiable fraction of OO is still unresolved, and new compounds are constantly being reported (35). Especially, in untargeted metabolic profiling studies many features remain unidentified or misidentified due to the lack of valid databases, the unavailability of reference standards even for major constituents, and the limitations of analytical techniques used (36). Particularly for LC-MS based metabolic profiling approaches, the analyzer is critical for the accurate identification of the detected features and accurate interpretation of results (37). As a consequence, the need for the development of robust and reliable analytical strategies for ensuring OO quality and authenticity has risen as a priority issue for consumers, suppliers, and regulatory agencies.

Among these lines, Greece is the country possessing the highest OO consumption (38) and is the third OO production country globally. Several approaches have been suggested since the 1980s based either on glycerol and later biophenols fraction, using different experimental protocols and analytical techniques (39–43). Additionally, several studies have been carried out for the classification of OOs coming from different Greek olive tree varieties (31, 44–46). To the best of our knowledge, there are limited studies focusing on the most widespread Greek olive variety, Koroneiki, and only two studies have been published for the influence of the employed cultivation practice in Greek OOs (47, 48). In addition, there is only one study investigating the effect of the production procedure on composition (49). Both of the aforementioned factors have been found to influence OO quality (50, 51), but until now they have not been well-studied in Greek OOs. Moreover, no previous study exists to investigate origin, cultivation, and production procedure at the same time or to study simultaneously both chemical parts (glycerol and biophenols) of OO.

Hence, the aim of the current study was to develop a fast and robust methodology for the classification as well as quality and authenticity assessment of Koroneiki Greek EVOOs according to their geographical origin, cultivation practice, and production procedure system. Additionally, it aims at using untargeted metabolic profiling concepts combined with dereplication methods to reveal specific compounds and chemical classes as markers for these quality parameters, for both glycerol and biophenols fraction of OO, taking advantage of the excellent identification competence of two FT analyzers. Flow Injection Analysis-Magnetic Resonance Mass Spectrometry (FIA-MRMS) with remarkable accuracy, resolution, and speed was integrated with a UPLC-HRMS/MS (orbitrap analyzer) platform providing HRMS/MS data. Thus, using FIA-MRMS, a rapid methodology of 2 min for intact oil and 8 min for biophenols, per sample was developed and applied. Data were processed using MDA and distinct clusters were revealed associating certain metabolites with olive tree geographical origin, production procedure, and cultivation practice employed. To our knowledge, this is the first time that certain marker compounds and classes abundant in Greek EVOOs are associated with three quality parameters employing at the same time FIA method and the identification confidence of MR analyzer.

## Materials and Methods

### Samples Collection and Registration

Samples were obtained directly from OO producers and cooperatives during the harvesting period 2015–2016 from three producing areas of Greece. Only samples from Koroneiki variety were collected, being the most established cultivation in Greece (60%). During sample collection, complete information accompanying each sample (metadata) was thoroughly assembled, i.e., production procedure, cultivation practice, exact location of olive grove, ripening stage, olive oil storage, and the categorization of olive oil based on the free acidity index (Table A1 in Supplementary Material). The selection of the geographical regions was based on a previous study of our group, investigating the quantitative profile of certain biophenols in Greek EVOOs (52). Over 300 monovarietal EVOO samples were initially collected from Crete, Peloponnese, and Ionian islands, representing three OO production regions, three production procedures, and three cultivation practices. Ultimately, after the implementation of certain acceptance criteria the number of samples was narrowed down to 208 (see section Samples Selection and Registration) (Table 1). After collection, all EVOOs were directly centrifuged and stored under nitrogen conditions in dark, glassy vials at −20°C to retain their chemical stability during storage (53).

**Table 1:** Representation of the total number of samples under investigation (*n* = 208).

**Quality parameters**	**Subclasses**	**Number of samples**
Geographical origin	Peloponnese	89
	Crete	85
	Ionian islands	34
Production procedure	Two phase	117
	Three phase	87
Cultivation practice	Conventional	104
	Integrated	38
	Organic	45

*Samples are grouped in three quality parameters: Geographical origin (n = 208), Production procedure (n = 204), and Cultivation practice (n = 187). Quality parameters are divided in subclasses and the respective numbers of samples are tabulated*.

### Biophenols Extraction

OO samples were extracted following the IOC proposed protocol (54) with minor modifications for process accelerating purposes (52). In brief, 1 g (±0.001) of EVOO was weighted and dissolved in 1 mL of *n*-hexane (*n*-hex). The solution of oil and *n*-hex was mixed and homogenized with MeOH/H_2_O 80:20 (v/v) using a vortex mixer for 3 min and then centrifuged in 3,000 rpm for 3 min for phases separation. The same procedure was repeated twice. The extracts were defatted twice with *n*-hex and evaporated under vacuum conditions and centrifugation at 30°C (GeneVac HT-4X). After evaporation, the dried extracts were stored in glassy vials at −20°C until analysis.

### Total Phenolic Content (TPC) Determination via Colorimetric Assay

As a widely used parameter Total Phenolic Content (TPC) was also evaluated for the derived extracts via Folin-Ciocalteu colorimetric assay (or gallic acid equivalence method). Gallic acid calibration curve was plotted by preparing eight different concentrations of gallic acid (2.5, 5, 10, 12.5, 20, 25, 40, and 50 μg/mL) mixed with Folin-Ciocalteu reagent (ten-fold diluted) and sodium carbonate solution (7.5% w/v). Extracts were prepared in the appropriate dilution with the same reagents and analyzed in triplicates. The absorption was measured at 765 nm. TPC values were expressed as mg of gallic acid equivalent/Kg of EVOO using the resulted standard calibration curve of gallic acid (*R*^2^ = 0.9982) (Figure A1 in Supplementary Material). The detailed results are presented in Table A1 in Supplementary Material.

### FIA-MRMS Analysis

MRMS analysis was employed for both intact EVOOs samples and their corresponding biophenols extracts. Flow injection analysis (FIA) coupled to solariX 7T MRMS system (Bruker Daltonik GmbH, Bremen, Germany) was used. EVOO samples were dissolved 1:1,000 in 50:50 CH_2_Cl_2_:MeOH containing 10 mM ammonium acetate. Spectra were acquired in the mass range of 147–3000 *m/z* with a resolving power of 450,000 at *m/z* 400. Spectra were externally calibrated with NaTFA cluster and additionally internally calibrated with a lock mass of deprotonated palmitic acid at *m/z* 255.23295. Mass spectra were obtained in negative ion mode with 24 scans per spectrum and the acquisition time was 0.75 min. A sample loop was filled with 20 μL sample solution and transferred to the electrospray ionization source (ESI). During spectra acquisition the flow was 10 μL/min. The total injection time was 2 min. Five repetitive measurements were performed for each sample.

The corresponding biophenols extracts were diluted 1:20 in 50% MeOH with 10 mM ammonium acetate. Spectra were acquired in the mass range of 107–3,000 *m/z* with a resolving power of 300,000, at *m/z* 400. Spectra were externally calibrated with NaTFA cluster. Mass spectra were obtained in negative mode with 92 scans per spectrum and the acquisition time was 6 min. A sample loop was filled with 100 μL sample solution and transferred to the ESI. During spectra acquisition the flow was 10 μL/min. The total injection time was 8 min. Three repetitive measurements were performed for each sample.

### UPLC-HRMS and HRMS/MS Analysis

UPLC-ESI-HRMS and high resolution tandem MS (HRMS/MS) were employed only for the analysis of biophenols extracts. H class Acquity UPLC system (Waters, USA) coupled to a LTQ-Orbitrap XL hybrid mass spectrometer (Thermo Scientific, USA) was used. The extracts as well as the quality control (QC) pooled sample were prepared in the final concentration of 500 μg/mL diluted in 1:1 MeOH:H_2_O. For the separation, H_2_O with 0.1% formic acid (FA) was used as solvent A and acetonitrile (ACN) as solvent B. The elution method started with 2% of B and in 2 min reached 21%. In the next 4 min the percentage of B increased to 44.5%. Finally, at 9 min, B reached 100% and was maintained for 2 min. The next minute, the system returned to the initial conditions and stayed for 3 min for system equilibration. A Thermo Hypersil Gold C-18 (50 mm × 2.1 mm, 1.9 μm) column was used for the separation, with a stable temperature of 40°C. The measurements were performed with a total acquisition time of 15 min and a flow rate of 400 μL/min. The injection volume was 10 μL and the auto sampler temperature was 7°C. Mass spectra were obtained in negative ion mode using ESI. The capillary temperature was set at 350°C, capillary voltage at −10 V, and tube lens at −40 V. Sheath and auxiliary gas were adjusted at 40 and 10 arb, respectively. Mass spectra were recorded in full scan mode in the range of 115–1,000 *m/z*, with resolving power 30,000 at 500 *m/z* and scan rate 1 microscan per second. HRMS/MS experiments were obtained in data-depending method with collision energy 35.0% (*q* = 0.25). The system was calibrated externally every 50 injections. Two repetitive measurements were performed for each sample.

### Data Processing and Chemometrics

MRMS data were recorded with ftmsControl 2.1 and processed with ProfileAnalysis 2.1. The mass lists of mass spectra were imported as feature lists based on the average mass spectrum of all spectra. This list was exported as an asc file. UPLC-MS data were recorded with Xcalibur 2.2. Raw files (.raw, Thermo) were imported to MZmine 2.26 software for data processing. Peak list was generated with centroid selection algorithm. For chromatogram building of the generated mass list, 0.05 min was set as minimum time of span and 5 ppm for mass tolerance. Chromatogram deconvolution module was employed and spectra were processed with local minimum search algorithm using R package. The minimum retention time range was set at 0.1 min and peak width 0.05–0.7 min. Chromatograms were aligned and spectra were normalized regarding retention time with 0.005 min tolerance. Join align method that aligns detected masses using a match score, calculated based on the mass and detection time of each peak, was used. Finally, gap filing was implemented, using peak finder method.

Both peak lists were imported to SIMCA 14.1 (Umetrics, Sweden) software for statistical analysis. Mainly, Principal Component Analysis (PCA) and Orthogonal Partial Least Squares-Discriminant Analysis (OPLS-DA) were implemented for sample visualization and discrimination, while s-plots were built between groups for determination and identification of statistically significant metabolites responsible for the observed trends and classifications. For this purpose, *p*-values in PCA and Variable Importance in Projection (VIP) values of OPLS-DA models, which rank variable contribution, were estimated and evaluated. *p* < 0.05 and VIP scores >1 were considered as statistically significant. The generated models were evaluated for their R2 and Q2 parameters indicating the goodness of fit and the predictability, respectively. Only models with R2 values close to 1, Q2 values over 0.5, or models with lower R2 but close to Q2 value were accepted. Permutation test was also applied for further validation of the models. Similarly, only models that succeeded in the permutation test were used for data visualization and subsequent VIP calculations.

### Dereplication and Structure Elucidation Workflow

The first step was the *p*- and VIP values calculations (Table A2 in Supplementary Material). Lists with significant features from both platforms were created based on the analyzed samples, i.e., intact EVOO and biophenols for FIA-MRMS and only biophenols for LC-Orbitrap MS. Chromatographic (Orbitrap MS) and spectrometric features (MRMS, Orbitrap MS) were used for dereliction process. Specifically, the ultra-high resolution of the MRMS instrumentation (<1 ppm) and SmartFormula™ (SF) tool enabled the automatic, high confidence assignment of molecular formulae as basis for compound identification. The MRMS spectra were imported and processed in MetaboScape 4.0 (Bruker Daltonics). Using the T-Rex 2D algorithm possible adducts as well as isotopes were removed. The import was conducted with a delta *m/z* of only 1 mDa with maximum possible charge state of 1 and an intensity threshold of 10,00,000 that corresponds to S/N 2. Molecular formulas were automatically assigned using the SF algorithm, which also takes into account isotopic fine structure information. Known and expected compounds in analyzed samples were automatically annotated using custom AnalyteLists.

For LC-Orbitrap MS, chromatograms and their corresponding HRMS spectra (<2 ppm) were investigated. Extraction ion method was used in parallel with peak-to-peak selection affording the corresponding full scan spectra. Suggested Elemental Composition (EC) algorithm together with isotopic patterns and ring double bond equivalent (RDBeq) values were used for identification. Most importantly, HRMS/MS spectra contributed to the identification of significant features in combination with on-line databases.

Likewise, reference standards of phenolic compounds commercially available or not (isolated in-house) were used to ensure identification confidence as well as an in-house database of olive compounds (17, 35). HRMS and MS/MS spectra of representative uncommon reference standards used are given in Figures A2–A14 in Supplementary Material. All marker compounds were annotated according to Chemical Analysis Working Group of the Metabolomics Standards Initiative (MSI) (55) following 4 levels of identification confidence, i.e., level 1, “identified metabolites” for makers identified using chemical reference standards; level 2, “putative annotated compounds” based on spectral similarity with libraries; level 3, “putative characterized compound classes” based on spectral properties consistent with a particular class of organic compounds; and level 4, “unknown” discernible MS signal that can be reproducibly detected and quantified [Table 2; (56)]. The “unknown” features were excluded from VIPs lists.

**Table 2:** Identified metabolites in EVOO and biophenol extracts.

**No**	**Experimental *m/z***	**Elemental composition**	**Suggested molecule**	**Identification level**	**Geographical region**	**Production procedure**	**Cultivation practice**
1	119.034969	C_4_H_8_O_4_	2-(2-hydroxyethoxy)acetic acid	2	Peloponnese	Two-phase	Integrated
2	121.029503	C_7_H_6_O_2_	Benzoic acid	1	Peloponnese	n.c.^b^	Integrated
3	137.06081	C_8_H_10_O_2_	Tyrosol	1	Peloponnese	Two-phase	Integrated
4	143.107736	C_8_H_16_O_2_	Octanoic acid	2	Peloponnese	Two-phase	Integrated
5	153.01931	C_7_H_6_O_4_	Protocatehuic acid	1	Peloponnese	Two-phase	Integrated
6	153.055719	C_8_H_10_O_3_	Hydroxytyrosol	1	Peloponnese	n.c.^b^	Conventional
7	179.071361	C_10_H_12_O_3_	Tyrosol acetate	2	Ionian islands	Two-phase	Conventional
8	183.066297	C_9_H_12_O_4_	Dialdehydic form of decarboxymethyl elenolic acid	1	Ionian islands	Two-phase	Conventional
9	195.066278	C_10_H_12_O_4_	Hydroxytyrosol acetate	2	Peloponnese, Crete	Two-phase	Conventional
10	199.170347	C_12_H_24_O_2_	Lauric acid	2	Peloponnese	Two-phase	Integrated
11	225.076859	C_11_H_14_O_5_	Deoxy elenolic acid	2	Peloponnese	n.c.^b^	Conventional
12	227.201648	C_14_H_28_O_2_	Myristic acid	2	Peloponnese	Two-phase	Integrated
13	241.071755	C_11_H_14_O_6_	Elenolic acid	1	Peloponnese	Three-phase	Conventional
14	241.21729	C_15_H_30_O_2_	Pentadecanoic acid	2	Peloponnese	Two-phase	Integrated
15	253.217315	C_16_H_30_O_2_	Palmitoleic acid	2	Peloponnese, Ionian islands	Two-phase	Integrated, organic
16	255.087423	C_12_H_16_O_6_	Elenolic acid methyl ester	2	Peloponnese	Three-phase	Conventional
17	255.232958	C_16_H_32_O_2_	Palmitic acid	2	Peloponnese	Two-phase	Integrated
18	257.066694	C_11_H_14_O_7_	Hydroxylated form of elenolic acid	1	Crete, Ionian islands	n.c.^b^	Conventional
19	267.23293	C_17_H_32_O_2_	Margaroleic acid	2	Peloponnese	Two-phase	Integrated
20	269.045551	C_15_H_10_O_5_	Apigenin	1	Peloponnese	Three-phase	Conventional
21	269.103081	C_13_H_18_O_6_	Elenolic acid ethyl ester	1	Peloponnese.		Conventional
22	269.248578	C_17_H_34_O_2_	Margaric acid	2	Peloponnese	Two-phase	Integrated
23	279.23298	C_18_H_32_O_2_	Linoleic acid	1	Ionian islands	Two-phase	Organic
24	281.248634	C_18_H_34_O_2_	Oleic acid	1	Crete, Ionian islands	Two-phase	Conventional, Integrated
25	283.264257	C_18_H_36_O_2_	Stearic acid	2	Peloponnese	Two-phase	Conventional, integrated
26	285.040502	C_15_H_10_O_6_	Luteolin	1	Peloponnese	Three-phase	Conventional, integrated
27	295.227889	C_18_H_32_O_3_	Hydroxylinoleic acid	2	Crete	Two-phase	Integrated
28	297.243504	C_18_H_34_O_3_	Hydroxyoleic acid	2	Crete	Three-phase	Conventional, integrated
29	299.056138	C_16_H_12_O_6_	Luteolin-7 methyl-ether	2	Peloponnese	Three-phase	Conventional
30	299.20161	C_20_H_28_O_2_	PUFA,C-20^c^	3	Peloponnese	Two-phase	Integrated
31	299.259173	C_18_H_36_O_3_	Hydroxystearic acid	2	x^a^	Three-phase	Conventional
32	303.123779	C_17_H_20_O_5_	Oleocanthal	1	Crete	Two-phase	Organic, conventional
33	303.232928	C_20_H_32_O_2_	Arachidonic acid	2	x^a^	x^a^	Integrated
34	309.284317	C_20_H_38_O_2_	Gondoic acid	2	Crete	Three-phase	x^a^
35	311.295541	C_20_H_40_O_2_	Arachidic acid	2	Crete	Three-phase	Conventional, integrated
36	313.238436	C_18_H_34_O_4_	Octadecanedioic acid	2	Crete	n.c.^b^	Integrated
37	319.116536	C_17_H_20_O_6_	Oleocanthalic acid	1	n.c.^b^		x^a^
38	319.118731	C_17_H_20_O_6_	Oleacein	1	Crete		Organic, conventional
39	335.113652	C_17_H_20_O_7_	Hydroxylated derivative of decarboxymethyl oleuropein aglycon	2	Crete	Two-phase	Conventional, organic
40	339.232889	C_23_H_32_O_2_	Docosanoic acid	2	Peloponnese	Two-phase	Integrated
41	357.134425	C_20_H_22_O_6_	Pinoresinol	1	Peloponnese	Three-phase	Conventional
42	361.129246	C_19_H_22_O_7_	Aldehydic form of ligstroside aglycon	1	Peloponnese	Three-phase	Conventional, organic
43	367.361121	C_24_H_48_O_2_	Lignoceric acid	2	Crete	Three-phase	n.c.^b^
44	377.124147	C_19_H_22_O_8_	Aldehydic form of oleuropein aglycon	1	Crete	Three-phase	Conventional
45	389.290828	C_21_H_42_O_6_	SFA^c^	3	Ionian islands	Three-phase	Conventional
46	391.139885	C_20_H_24_O_8_	Methyl oleuropein aglycon	2	Crete	Three-phase	Conventional
47	393.119148	C_19_H_22_O_9_	Hydroxylated derivative of aldehydic form of oleuropein aglycon	2	Crete	Three-phase	Conventional
48	393.155482	C_20_H_26_O_8_	Hydroxylated derivative of ligstroside aglycon	2	Peloponnese	Three-phase	Conventional, organic
49	409.15044	C_20_H_26_O_9_	Oleuropein aglycon derivative	2	Crete, Peloponnese	Three-phase	Conventional
50	413.290878	C_23_H_42_O_6_	TG		Ionian islands	Two-phase	Organic
51	415.139887	C_22_H_24_O_8_	Acetoxypinoresinol	1	Peloponnese	Three-phase	Conventional
52	415.306484	C_23_H_44_O_6_	MUFA ester	3	Ionian islands	Two-phase	Organic
53	417.155523	C_22_H_26_O_8_	Syringaresinol	2	Peloponnese	Three-phase	Conventional
54	417.322155	C_23_H_46_O_6_	SFA ester^c^	3	Peloponnese	Two-phase	Conventional
55	423.422612	C_28_H_53_O_2_	Montanic acid	2	Crete	Two-phase	n.c.^b^
56	455.353111	C_30_H_48_O_3_	Oleanolic acid	1	Crete	Three-phase	Conventional
57	471.347941	C_30_H_48_O_4_	Maslinic acid	1	Crete	Three-phase	Conventional
58	611.525847	C_37_H_72_O_6_	DG derivative	3	Ionian islands	Two-phase	Organic
59	629.491862	C_37_H_70_O_5_Cl	DG	3	Ionian islands	Two-phase	Conventional, Organic
60	637.541557	C_39_H_74_O_6_	Trilaurin	2	Ionian islands	Two-phase	Organic
61	653.542317	C_39_H_73_O_7_	DG derivative	3	Ionian islands	Two-phase	Organic
62	655.515738	C_39_H_72_O_5_Cl	DG	3	Ionian islands	Two-phase	Conventional
63	679.554723	C_41_H_75_O_7_	DG derivative	3	Peloponnese	Three-phase	Organic
64	849.763319	C_53_H_102_O_7_	TG derivative	3	Ionian islands	Two-phase	Organic
65	867.725262	C_53_H_100_O_6_Cl	TG	3	Ionian islands	Two-phase	Conventional
66	875.774536	C_55_H_104_O_7_	TG derivative	3	Ionian islands	Two-phase	Organic
67	890.714835	C_57_H_110_O_6_	Stearin	2	Ionian islands	Two-phase	Conventional
68	891.725417	C_55_H_100_O_6_Cl	TG	3	Ionian islands	Two-phase	Conventional, organic
69	893.742758	C_55_H_102_O_6_Cl	TG	3	Ionian islands	Two-phase	Conventional
70	895.732846	C_55_H_104_O_6_Cl	TG	3	Peloponnese	Two-phase	Integrated, conventional
71	914.753581	C_57_H_98_O_6_	Linolein	2	Peloponnese	Two-phase	Conventional
72	919.754312	C_57_H_104_O_6_Cl	Triolein	2	Ionian islands	Two-phase	Conventional

*All the metabolites are numbered according to the first column. Experimental m/z, elemental composition as well as identification level are given. Each metabolite is accompanied with the primary type of classID i.e., geographical region, production procedure, and cultivation practice*.

a *Metabolites marked with “x” are not found in the VIP list for the corresponding quality parameter*.

b *Indication of “n.c.” is referred to features that no conclusion could be made with due to the low number of samples*.

c *SFA, saturated fatty acid; PUFA, polyunsaturated fatty acid*.

## Results and Discussion

### Samples Selection and Registration

A critical factor in metabolomic approaches is sampling quality, by means of sample size as well as complete and accurate sample information (metadata). Comprehensive and precise metadata is the basis of data validity and ultimately results in soundness since correlations with certain parameters of chemical composition, concertation levels, and/or markers are anticipated. Generally, in edible oils and particularly in OO, this is a complex issue. The main reason is the difficulty to collect reliable samples; as in the current study, monovarietal EVOOs were selected for investigation and consequently the market was not a choice. For this reason, a commonly small number of samples is subjected for analysis, diminishing significantly the statistical confidence of the obtained results or leading to fragmented and incomplete conclusions (48). Despite the plethora of studies available, the quality of metadata is usually overlooked or underestimated.

In the current study, special attention has been given to the sampling and precise metadata as well as the selection of the discriminating parameters. Given the fact that Koroneiki variety dominates Greece (60% of total cultivations), only OO samples from the specific olive variety were selected for investigation from our in-house OO library. Koroneiki is considered as the most common and suitable tree cultivar for OO production in the south of Greece, due to its adaption to dry and hot climates and the production of small drupes, with high ratio of skin/flesh, generating high yields of oil (47).

Following variety, the second selection criterion was extra virgin index, since it is estimated that more than three-fourths of the produced Greek oils are EVOOs. Only EVOO samples were included while other OO qualities were excluded. Ripening stage was the third exclusion parameter. It is well-known that olive drupe maturity is strongly associated with the chemical composition of OO (57). Therefore, only EVOO samples characterized by close ripening stage (November–December) were selected. Finally, OO samples with incomplete metadata were excluded. Thus, from the 300 initially collected samples only 243 were qualified to be further scrutinized.

The number of samples was further reduced taking into account the geographical origin. The two main regions, Peloponnese and Crete, responsible for ~75% of the total production, were rationally selected as more representative. Each region was further divided into two subareas, i.e., Heraklion and Lasithi (Crete) and Messenia and Laconia (Peloponnese) based on the same rationale. Additionally, Crete and Peloponnese are the dominating OO producing areas of Greece and can consequently provide an adequate number of samples produced in different olive oil mills and with different cultivation practices. Considering that both origin and cultivation conditions are strongly related to climate conditions, it should be noted that both Crete and Peloponnese are characterized by high temperatures during the whole year and low precipitation levels. In order to explore in more detail these parameters, samples from Ionian islands (Cephalonia and Ithaca) were also included. Ionian islands are not an area of high OO production. However, these islands have a microclimate utterly different (low temperatures during the winter and high precipitation levels the whole year) from Crete and Peloponnese and can be a “negative” control in our study.

For cultivation practice, conventional, integrated, and organic farming were selected. Conventional practice is mainly used in Greece for olive tree growing, while integrated has started to become more popular. In these two practices, synthetic fertilizers and pesticides are used and the difference concerns that in the second there are limitations in terms of the quantity of fertilizers, chemicals, and propagation material. In organic farming, chemicals are forbidden and soil regeneration is based on physical processes (58, 59).

Regarding production procedure, two-phase, three-phase, and traditional mills were used for OO production. Nonetheless, samples produced with traditional pressing system were excluded from the analysis, due to the low number of samples. Nowadays, this kind of production is almost abandoned in Greece. Two-phase and three-phase centrifugation systems are the most common used methods and their difference lies on the amount of the added water during the step of oil separation from paste; two-phase centrifugators are more evolved and require lower amounts of added water in comparison to three-phase (51). These production procedures and cultivation practices are the most widely employed in Greece and in consequence disclose high interest for investigation. Samples coming from organic cropping that were not certified were also excluded. Overall, the number of valid samples to be forwarded for the analysis was finally 208. Table 1 illustrates the sample collection subjected to analysis.

### FIA-MRMS Analysis

Generally, GC-MS, LC-HRMS, and NMR are the most broadly performed approaches under metabolic profiling or fingerprinting concepts, for quality control of OO, characterized by different pros and cons (29, 30, 60). Numerous studies are available in the literature investigating fraudulence and authentication of OO using these methods (61). In the current work, MRMS analyzer coupled to FIA was employed taking advantage of analyzers' excellent resolution as well as the minimum analysis time offered by FIA. In parallel, the more established LC-ESI-Orbitrap MS analysis was also used for comparison purposes but also to increase identification confidence based on HRMS/MS spectra. Using FIA-MRMS, intact oil samples were analyzed as well as the biophenols extracts derived from the same samples. On the other hand, with LC-HRMS only biophenols samples were analyzed. Data derived from both platforms were subjected to MDA using unsupervised and supervised methods for the identification of chemical markers related to quality parameters of OO. To our knowledge there are limited studies available using FIA-MRMS for the analysis of OO, none combining two HRMS platforms and especially on Greek EVOOs.

#### Geographical Origin

As mentioned already, the quality and thus the safety as well as the beneficial health effects of OO depend on multiple factors such as olive cultivar, geographical origin, pedoclimatic conditions, agronomic practices, and processing methods. Therefore, the identification and annotation of EVOOs according to their origin, such as PDO, PGI, and traditional specialties guaranteed (TSG), have become essential information to ensure authenticity and consumer protection (6). Despite the efforts made in the area, a decisive and robust solution is still not available.

**Figure 2:**
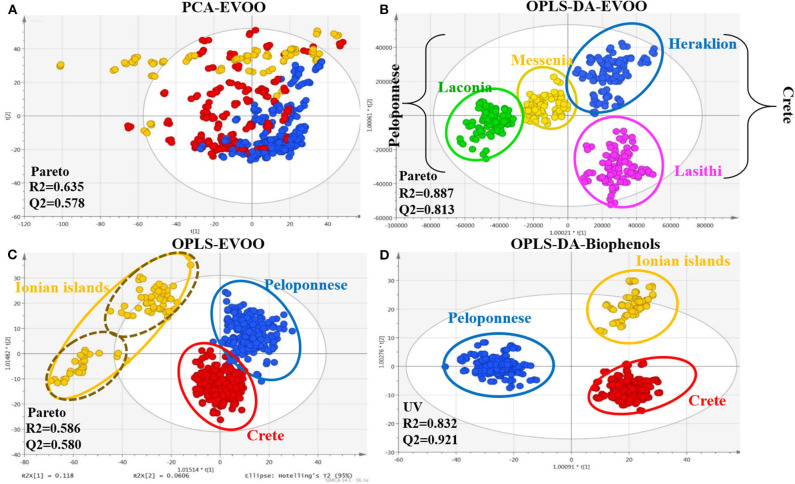
Scores plots of EVOOs and their corresponding biophenol extract acquired via FIA-MRMS. Observations are colored coded according to geographical origin. **(A)** PCA scores plot of EVOOs including all observations (pareto scaling); **(B)** OPLS scores plot of EVOOs including all observations (pareto scaling); **(C)** OPLS-DA plot of EVOOs excluding Ionian island samples (pareto scaling); and **(D)** OPLS-DA scores plot of biophenol extracts including all observations (UV scaling). R2/Q2 model parameters are annotated (bottom-left).

Hence, all 208 monovarietal EVOO samples, both intact EVOO and biophenols extracts, were analyzed with FIA-MRMS. It is important to note that a method of 2 and 8 min, respectively, including equilibration and MS stability time, was developed and applied to shrink the analysis time and minimize prior sample preparation. The spectra were subjected to data processing prior to MDA. Pareto scaling provided the best visualization and fitting results for intact EVOO, while unit variance scaling (UVS) was used for the corresponding biophenols extracts. From the EVOO dataset, 2,637 variables (*m/z*) were revealed, and 5,079 were revealed from the biophenols dataset. All the generated models, using supervised methods (OPLS, OPLS-DA), succeeded in validation tests, holding high scores for the R2 and Q2 parameters. Figure 2 illustrates representative scores plots using geographical origin as class parameter.

In PCA scores plot of intact EVOOs (Figure 2A), an unexpected clear tendency is observed separating the samples into three basic areas; Peloponnese (blue), Crete (red), and Ionian islands (yellow). The majority of outliers (outside 95% confidence Hotelling range) are originated from Ionian islands followed by samples of Crete. Interestingly, based on metadata, most of the outliers correspond to Ithaca island. In OPLS scores plot (Figure 2B), well-defined groups are revealed with acceptable fitting parameters. Especially for Ionian islands, two subgroups are clearly evident representing the two different collection islands, i.e., Cephalonia (upper group) and Ithaca (lower group). Likewise, outliers correspond to samples from Ithaca island. It is worth noting that the two islands are geographically very close, indicating the power and the sensitivity of the model.

Additionally, a distinct separation between the groups of Peloponnese/Crete and Ionian group is observed, indicating the chemical discrepancy of Ionian samples in comparison to the other two areas. Excluding Ionian islands samples and using OPLS-DA method (Figure 2C), higher fitting parameters and clear clusters are disclosed. Interestingly, there is a distinct separation between the two basic areas, Peloponnese and Crete, on the first component (40% explained variation) and apparent separation among the geographical subareas at the second component (26% explained variation). For biophenols only the OPLS-DA model passed the validation process (permutation test and acceptable fitting parameters). Distinct grouping of the three basic areas is attained (Figure 2D). Once more, Ionian islands are characterized by a number of outliers coming from Ithaca.

Following the scores plots, the corresponding loadings plots were thoroughly examined (Figure A15 in Supplementary Material) for the identification of compound classes and marker metabolites, responsible for the observed classification and correlation with geographical origin. VIP values from the OPLS-DA models in pairs were used to prioritize the detected variables. With VIP >1, 297 features for EVOOs and 445 for biophenols extracts were determined. Using data from MRMS and Orbitrap MS and following the dereplication work-flow (section Dereplication and Structure Elucidation Workflow), the statistically significant identified compounds are presented in Table 2.

**Figure 3:**
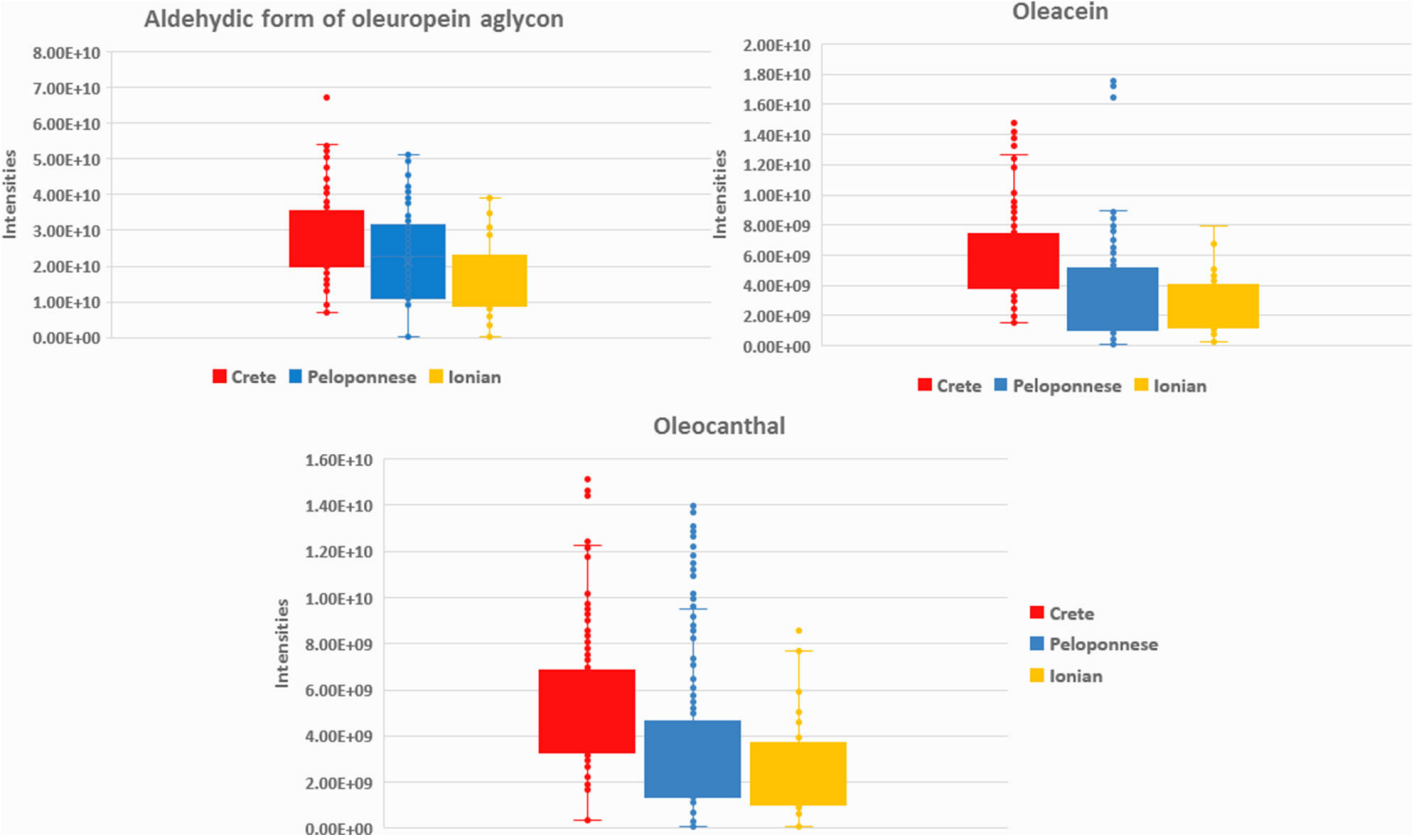
Box-plots of aldehydic form of oleuropein aglycon (**44**), oleacein (**38**), and oleocanthal (**32**) in the three basic geographical regions Crete (red), Peloponnese (blue), and Ionian islands (yellow).

In total, 72 metabolites were identified both in EVOOs and biophenols extracts as geographical origin markers. The identification results denote that each geographical region is characterized by certain chemical categories and compounds indicating their chemical discrepancy. Based on Table 2 (and Table A2 in Supplementary Material), EVOOs from Crete are mainly characterized by secoiridoid derivatives. Specifically, aldehydic form of oleuropein aglycon (**44**) is present in almost all samples from Crete, revealing the highest relative levels among all groups (VIP = 14.02). Also its derivatives, i.e., hydroxylated aldehydic form of oleuropein aglycon (**47**), hydroxylated decarboxymethyl oleuropein aglycon (**39**), oleacein (**38**), and oleocanthal (**32**), are characteristic secoiridoid markers of Crete (Figure 3). Other compounds strongly correlated are triterpenic acids [maslinic acid (**57**), oleanolic acid (**56**)] and long chain fatty acids [lignoceric acid (**43**), montanic acid (**55**), gondoic acid (**34**), arachidic acid (**35**)]. It is useful to highlight here that triterpenic acids are also relatively polar constituents with important biological properties and are extracted with polyphenols from OO. It is noteworthy that oleic acid (**24**) is clearly present in higher levels in Crete and Ionian samples in contrast to Peloponnese as it is shown in metabolite-based projection plot (Figure A17 in Supplementary Material). Palmitic acid (**17**) follows the same trend, but the difference between the samples is not so evident. Based on bibliographic data, palmitic acid is formed from oleic acid (62), explaining the same trend in the two plots. These observations are in agreement with previous studies using classical methods for FAs determination verifying our approach (63).

**Figure 4:**
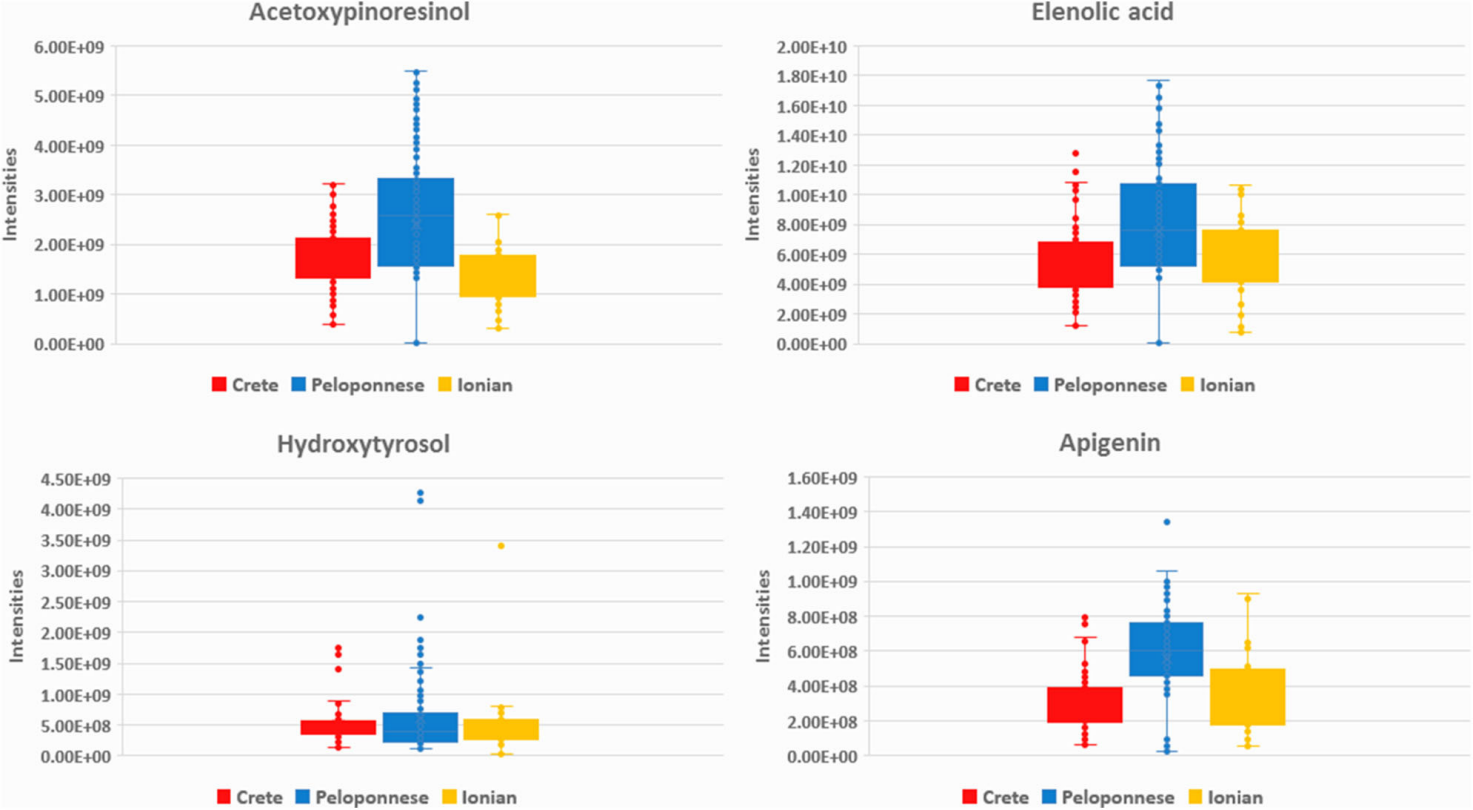
Box-plots of acetoxypinoresinol (**51**), elenolic acid (**13**), hydroxytyrosol (**6**), and apigenin (**20**) in the three basic geographical regions Crete (red), Peloponnese (blue), and Ionian islands (yellow).

In the case of Peloponnese, a different pattern is revealed. More chemical groups are represented, i.e., flavonoids [apigenin (**20**), luteolin (**26**), luteolin-7-methyl ester (**29**)], lignans [acetoxypinoresinol (**51**), pinoresinol (**41**), syringaresinol (**53**)], phenyl acids [protocatehuic acid (**5**), benzoic acid (**2**)], phenyl alcohols [hydroxytyrosol (**6**), tyrosol (**3**), and hydroxytyrosol acetate (**9**)] (Figure 4). It is worth noting that some compounds, e.g., lignans from the aforementioned groups, have been previously suggested as Koroneiki variety markers (64). Moreover, FAs such as pentadecanoic acid (**14**), palmitoleic acid (**15**), margaric acid (**22**), lauric acid (**10**), margaroleic acid (**19**), stearic acid (**25**), octanoic acid (**4**), and typical TGs [triolein (**71**) and linolein (**70**)] are characteristic of Peloponnese EVOOs. Regarding secoiridoids, elenolic acid, and its derivatives [elenolic acid methyl ester (**16**), elenolic acid ethyl ester (**21**), deoxyelenolic acid (**11**)] are found as the most statistically significant markers (Figure 4). In contrast to Crete, less complex secoiridoids, missing the phenylethanoid group, were identified with the exception of aldehydic form of ligstroside aglycon (**42**). Interestingly, high abundance of oleocanthal (**32**) was found in some specific samples (Lakonia subarea) even if it is not a statistically significant marker of Peloponnese EVOOs. In these cases, maybe other parameters, such as altitude, are responsible for these high levels. In a previously published study, authors claim that the discrimination between Lakonia and Crete is difficult, due to the same microclimatic conditions in these areas (high temperatures and low precipitation levels), associating their similar chemical profile with certain biosynthetic pathways (41).

Contrary to the other areas, Ionian island samples uncover completely different patterns. FAs dominate while biophenols levels are significantly low. Linoleic acid (**23**) derives as the most statistically significant marker of this region (Figure A18 in Supplementary Material), while its levels in the other regions are low. Oleic acid (**24**), which is considered as a predominant FFA of Koroneiki variety, retains its high levels. This fact can be explained from the activity of the enzyme oleate desaturase, which has been found to convert oleic acid to linoleic (65). Among the factors that regulate oleate desaturase activity is temperature (66). It has been recently reported that low temperatures lead to higher content of C18:2 and C18:3 FA to maintain the fluidity of the membranes (67). Also it has been reported that higher temperatures increase oleic acid content in OO and its percentages depends on the mean environmental temperatures during fruit growth (68). The high content of linoleic acid in Cephalonia has also been reported in another study where the authors calculated the FFA composition of different cultivars and locations of Ionian islands (43).

Moreover, Ionian samples are mainly characterized by DGs and their derivatives. These findings are in accordance with previous results of our group confirming the significant chemical differences of Ionian OO with OOs produced in other regions of Greece (52). The above findings may be explained by the different climate conditions in the three basic areas and the different adaption-behavior of Koroneiki variety in these microclimatic conditions. It has been generally proved that phenolic compounds content is being increased with the exposure to UV light and high temperatures (69). The environmental stress of Crete and Peloponnese seems to lead to higher production of biophenols in comparison to Ionian islands. In parallel, the relative lower temperatures of Ionian islands influence FFA composition and the saturation degree of OO (67). Despite the clear markers for each region under study, some compounds are found significant for two regions, simultaneously but with different VIP values. For instance, oleic acid is important for the separation of Crete and Ionian samples from Peloponnese, and palmitoleic acid could be used to differentiate Peloponnese and Ionian EVOOs from Cretan ones.

**Figure 5:**
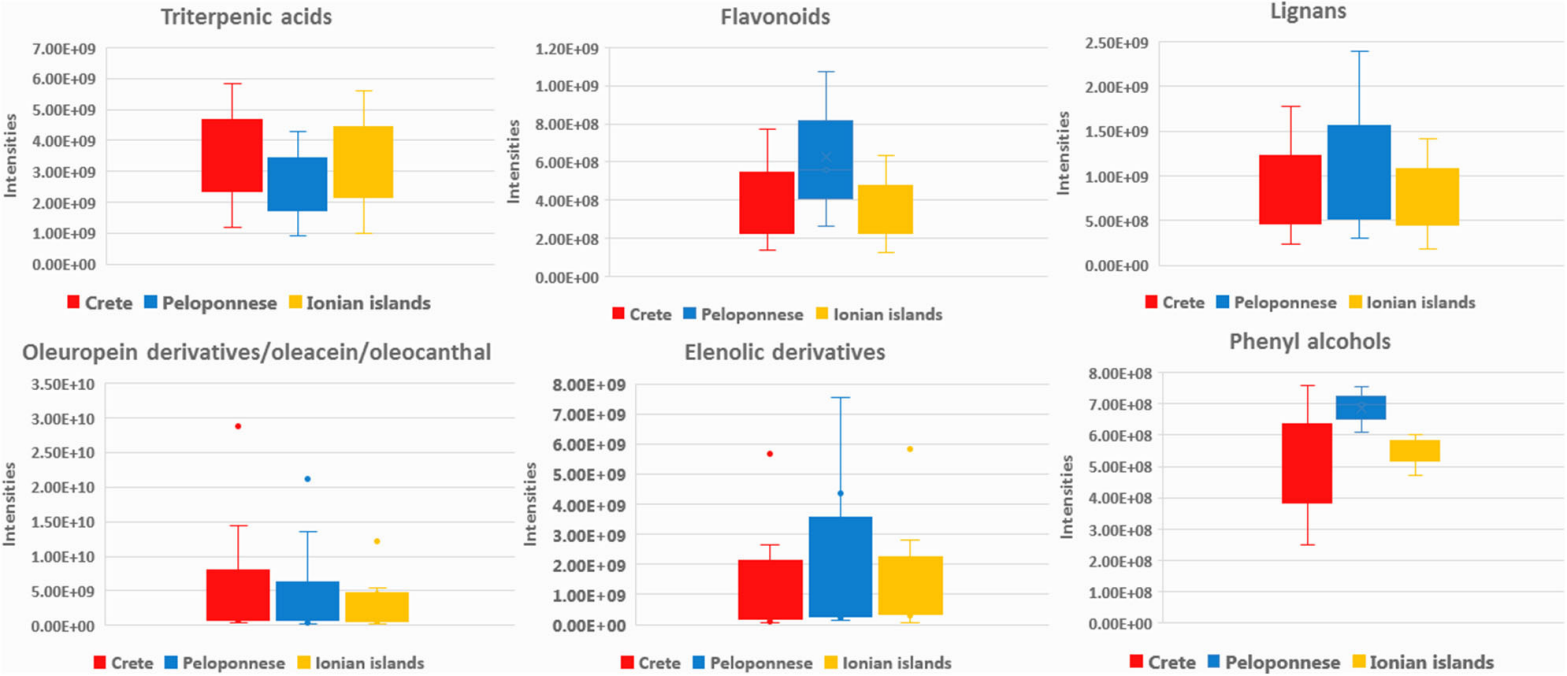
Box-plots representing intensities of statistical significant compounds grouped according to their chemical class; triterpenic acids, flavonoids, lignans, secoiridoids (oleuropein derivatives, oleacein, and oleocanthal), and elenolic acid derivatives as well as phenyl alcohols. Box-plots are presented according to geographical origin: Crete (red), Peloponnese (blue), and Ionian islands (yellow).

Summarizing the above findings and interpreting them by means of chemical classes, it is shown that OO from Crete (Heraklion and Lasithi) is characterized by higher levels of secoiridoids, triterpenic acids, long chain FAs, as well as increased levels of oleic and palmitic acid. On the other hand, Peloponnese OOs are more variable and richer in flavonoids, lignans, phenolic acids, elenolic acid derivatives, phenyl alcohols, as well as short chain FA. Finally, Ionian OOs are relatively poor in biophenols but with higher levels of FAs and DGs as well as linoleic acid (Figure 5).

#### Cultivation Practice

**Figure 6:**
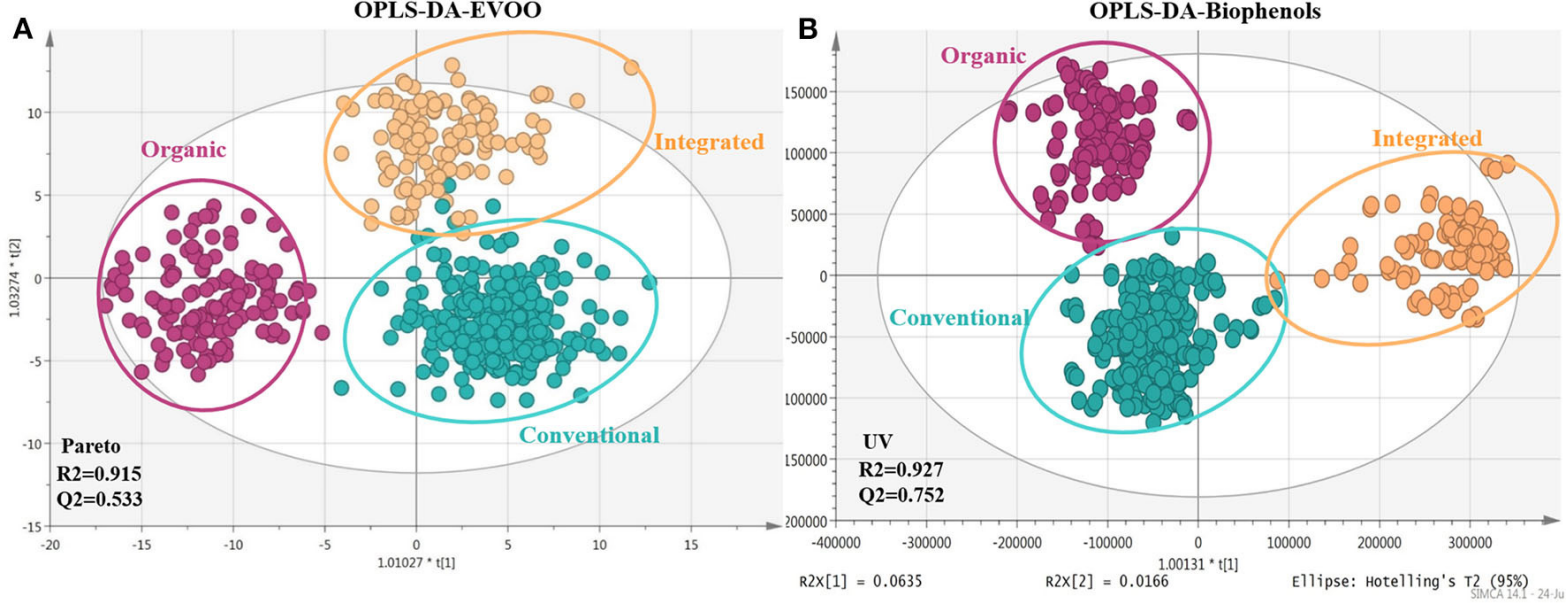
Scores plots of EVOOs and their corresponding biophenol extracts acquired via FIA-MRMS. Plots include the total number of observations and are colored coded according to cultivation practice classID; conventional (blue), integrated (light orange), and certified organic practices (magenta). **(A)** OPLS-DA scores plot of EVOOs with pareto scaling; **(B)** OPLS-DA scores plot of biophenols with UV scaling. R2/Q2 model parameters are annotated (bottom-left).

Agronomical practice used for the cultivation of the olive tree is a critical parameter affecting the composition of olive drupes and consequently the produced OO (50). In the current study, three basic farming practices were investigated, i.e., conventional, integrated, and certified organic. In total, 309 variables were found with VIP >1 for EVOOs and 429 for EVOO biophenols. In Figure 6A clear clustering between integrated and conventional practice is observed when intact oil analysis data are visualized. It is interesting that integrated and conventional samples, which apply similar maintenance and fertilization practices, are separated from organic farming on PC1 while integrated and conventional on PC2. On the other hand, in biophenols scores plot (Figure 6B) the clusters were tighter with higher R2/Q2 and better permutation test values (Table A3 in Supplementary Material) and regarding classes the samples of integrated cultivation are clustered separately to organic and conventional, suggesting closer composition into the latter. This observation possibly implies that cultivation method affects differently the glycerol and biophenols parts.

Investigating the loadings plots for intact EVOO (Figure A19 in Supplementary Material) and the statistically significant features, we observed a prominent accumulation of FFAs in integrated practice, followed by conventional. Especially, oleic acid was found in significant levels as well as TGs, in contrast to organic farming where mostly DGs were encountered. Interestingly, there is an influence on OO of integrated practice, which application is rapidly growing and is poorly investigated. There are studies available focusing mainly on FFAs (70), which compare organic vs. non-organic methods and most of the times investigating Spanish cultivars (65, 66). For Koroneiki, at least to our knowledge there is only one study comparing conventional vs. organic, which reports that only palmitoleic and oleic levels vary according to cultivation, however without including TGs and DGs (47).

**Figure 7:**
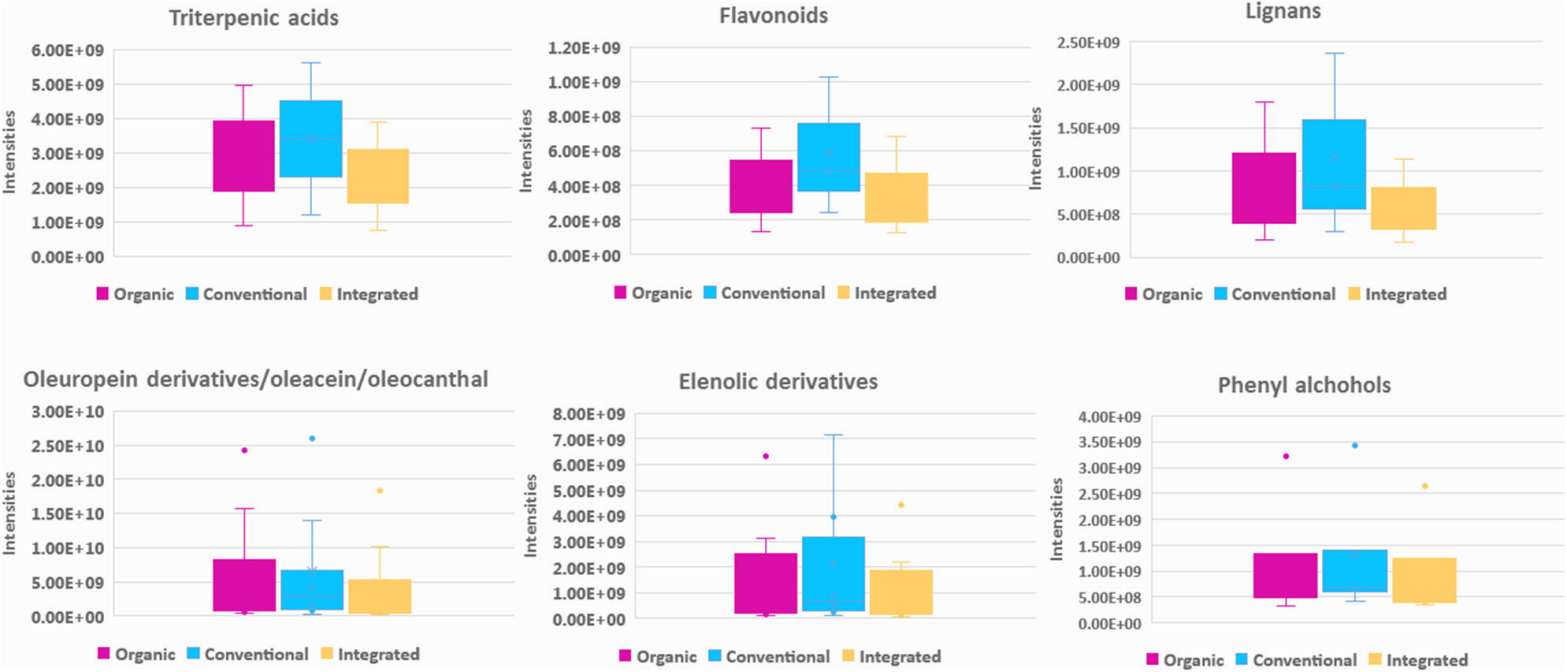
Box-plots representing intensities of statistical significant compounds grouped according to their chemical class; triterpenic acids, flavonoids, lignans, secoiridoids (oleuropein derivatives, oleacein, and oleocanthal) (elenolic derivatives), and phenyl alcohols. Box-plots are presented according to cultivation practice: organic (magenta), conventional (blue), and integrated (light yellow).

Regarding polar constituents, markers for conventional farming are compounds belonging to the majority of biophenols classes such as elenolic acid derivatives, phenylalcohols, lignans, flavonoids, as well as triterpenic acids (Figure 7 and Figure A21 in Supplementary Material). For integrated method, marker compounds are only certain phenolic acids, i.e., protocatechuic and benzoic acid. On the other hand, in organic practices we did not identify many biophenols as marker compounds. However, in a considerable number of samples characteristic secoiridoids and more specifically the relatively more polar were identified in higher levels in comparison to conventional practice, i.e., oleacein (**38**), oleocanthal (**32**), aldehydic form of ligstroside aglycon (**42**), and its derivatives as well as some aldehydic form of oleuropein aglycon derivatives (Figure 7). This observation initially seems in contrast with most of available studies reporting that organic practice produces higher biophenols levels, however measured as TPC values (47, 50, 71, 72). It is generally accepted that biophenols levels interrelates to the amount of the available water. Excess of water (usually provided to conventional practice) leads to decreased production of phenolic compounds (73), due to lower activity of L-phenylalanine ammonia lyase enzyme in olive fruits, which is the key enzyme in phenolics biosynthesis (74).

In order to get better insight, we proceeded with the measurement of TPC values in the analyzed samples. Indeed, as existing information suggests organic method showed higher TPC values followed by integrated and then conventional (Table A1 in Supplementary Material and Figure A20 in Supplementary Material). Based on both observations, it could be hypothesized that samples from organic cultivation contained higher levels of certain biophenols, i.e., polar secoiridoids even if conventional and integrated are characterized by more biophenols chemical classes. Thus, their determination could be exploited as an additional and more accurate indicator of organic cultivation derived OO compared to TPC, which is a general parameter and highly non-selective. It should be also noted that these markers appear in high concentrations generally in Greek EVOOs (52) and consequently encompass a high percentage of total polyphenols fraction.

Overall, using FIA-MRMS, it is possible to discriminate conventional, integrated, and organic method based on marker compounds and/or compounds classes in Greek EVOOs derived from Koroneiki variety. Specifically, for Koroneiki and integrated method, data are presented here for the first time.

#### Production Procedure

**Figure 8:**
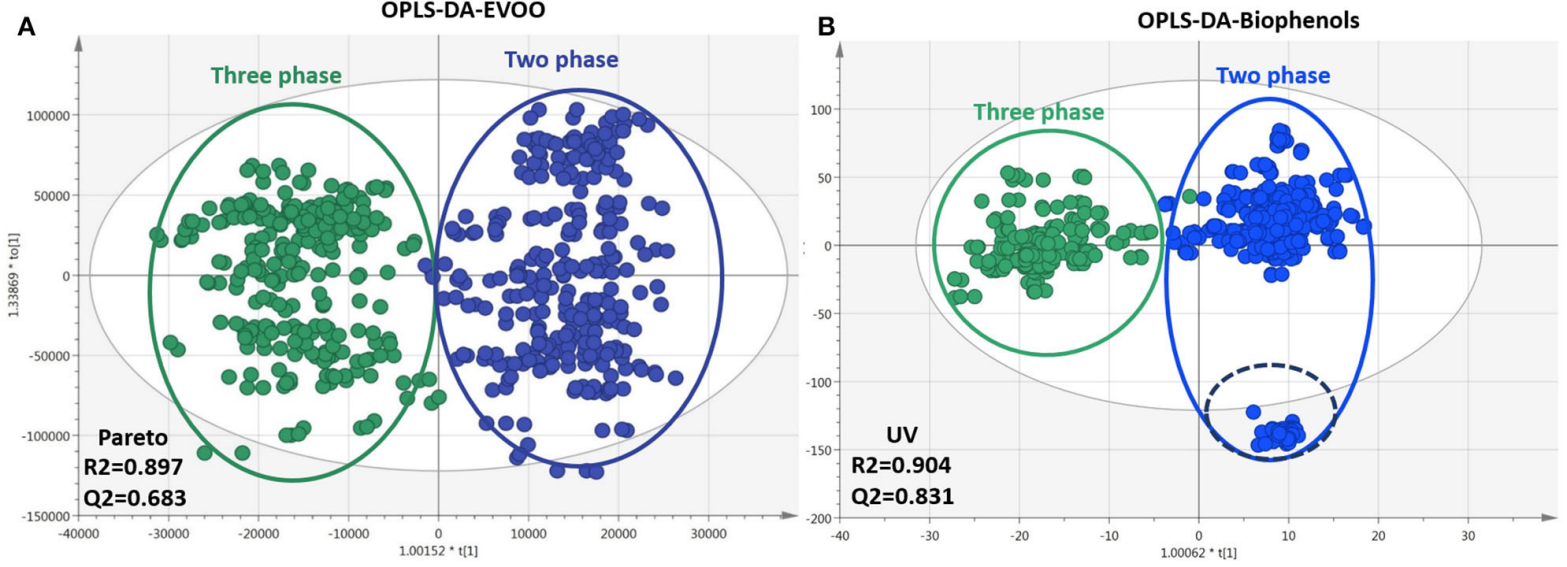
Plots of EVOOs and their corresponding biophenols extracts acquired via FIA-MRMS. Plots include the total number of observations and are colored according to production procedure classID: three phases (green), two phases (blue). **(A)** OPLS-DA of EVOOs treated with pareto scaling. **(B)** OPLS-DA of biophenols treated with UV scaling. R2/Q2 model parameters are annotated (bottom-left).

As mentioned already, two methods (centrifugation systems) are mainly used for the production of OO. In some cases, OO derived from traditional systems can be found, but their use is rather limited nowadays. Thus, EVOOs from traditional mills were not included in the study due to their low number (section Samples Selection and Registration). Similarly to the former classes, OPLS-DA models for EVOOs and biophenols, respectively, were generated and are presented in Figure 8. In total, 309 features for EVOOs and 429 for biophenols with VIP >1 were determined, ranked, and identified [in Figure A22 (Supplementary Material) the corresponding s-plot is presented]. In both plots a clear discrimination between the two different procedures is observed on PC1, while the model of biophenols showed higher fitting parameters R2/Q2, better permutation test results (Table A3 in Supplementary Material), and tighter clusters in comparison to EVOOs model (Figure 8). Concerning intact EVOO (Figure 8A), TGs, DGs, and FFAs were identified in both production systems without showing any particular trend, which is also verified from the model's characteristics such as observations diffusion and low fitting scores. In a previous study, authors describe that FAs are not influenced by production methodology (49), and therefore they can be regarded as unsuitable markers. Our results verify these findings and additionally show that TGs and DGs are not influenced and therefore cannot be used as marker class.

**Figure 9:**
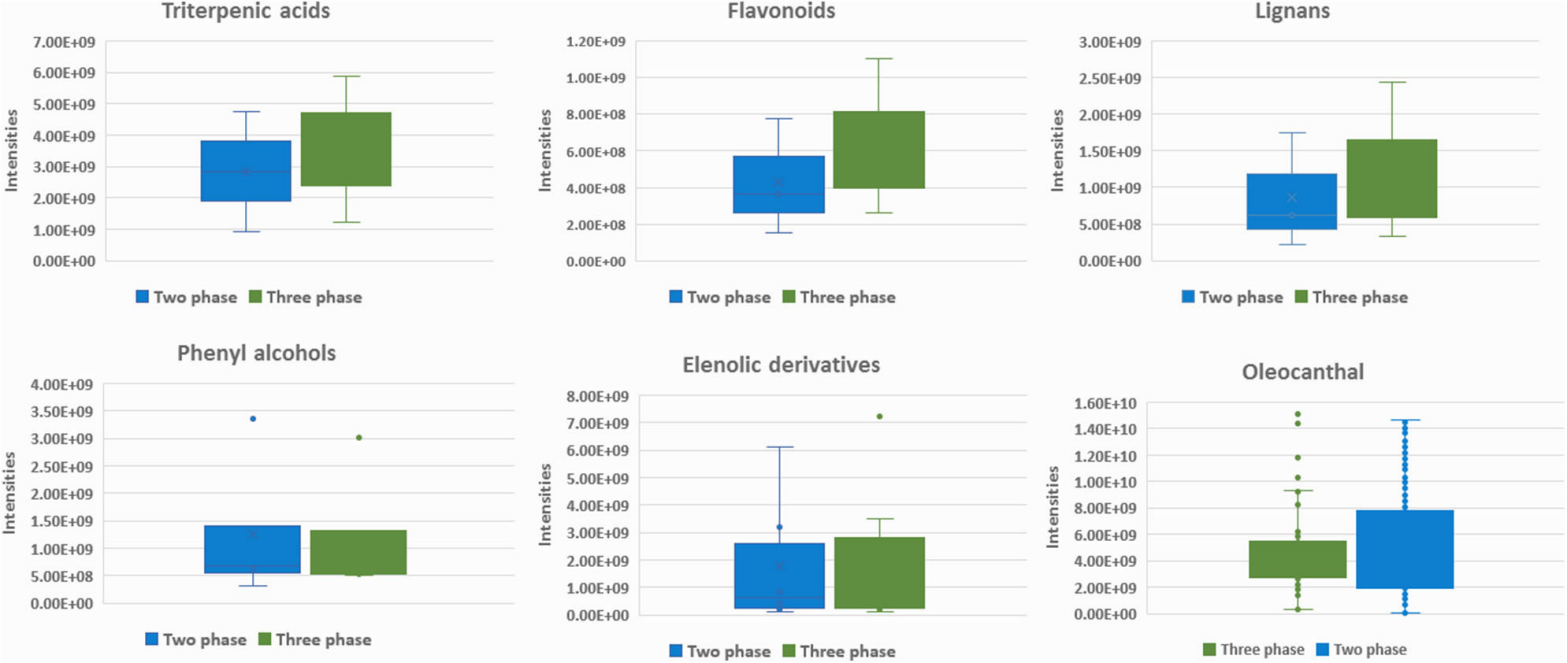
Box-plots representing intensities of statistical significant compounds grouped according to their chemical class: triterpenic acids, flavonoids, lignans, elenolic derivatives phenyl alcohols, and oleocanthal (**32**) from secoiridoids class. Box-plots are presented according to production procedure system: two phase (blue) and three phase (green).

For biophenols (Figure 8B) the observed pattern is different, and the discrimination between the two groups was much better as well as the validation scores. In specific, hydroxytyrosol (**6**), tyrosol (**3**), hydroxytyrosol acetate (**9**), tyrosol acetate (**7**), and the secoiridoids oleacein (**38**), oleocanthal (**32**), and oleocanthalic acid (**37**) were found statistically significant markers of two-phase milling (Figure 9). On the other hand, other secoiridoids, lignans, flavonoids, and triterpenic acids are more present in three-phase systems (Figure A23 in Supplementary Material). Furthermore, from TPC calculation, two-phase samples were found higher in polyphenols compared to three-phase samples (Table A1 in Supplementary Material and Figure A24 in Supplementary Material). These results suggest that the centrifugation system used for OO production affects not only the total levels of polyphenols but also its composition with two-phase system favoring the more polar phenolics. Our results are in agreement with previous studies indicating that OOs produced in two-phase mills contain higher phenolic content in comparison to three-phase due to the removal of phenols into waste during the addition of water in the malaxation stage (51). Moreover, one study also in Koroneiki variety reports that two-phase decanters resulted in OOs with higher content in phenyl alcohols and phenolic acids (49). In addition, in the current study we showed that also secoiridoids and specifically the more polar ones are marker compounds for two-phase systems while flavonoids and lignans could be indicative markers for three-phase produced OO.

It is interesting to note that in the two-phase samples group (Figure 8B), a subgroup of outliers (outside 95% confidence range) is observed. Investigating the metadata, we found that all the samples come from the same oil production plant in Lakonia (Peloponnese) but from different OO producers. Nevertheless, they are not grouped together when geographical origin is the class parameter (section Geographical Origin). These samples, in contrast to the rest of the samples, are characterized by phenolic acids [protocatechuic acid (**5**), benzoic acid (**2**)] and FFAs [palmitic acid (**17**), myristic acid (**12**), pentadecanoic acid (**14**), palmitoleic acid (**15**), margaric acid (**22**), lauric acid (**10**), margaroleic acid (**19**), stearic acid (**25**), octanoic acid (**4**)]. The reason is not clear. Although a different treatment, for example, uncommon malaxation temperature or time, has been possibly implemented during production and consequently influenced their chemical profile with the decrease of secoiridoids, which are marker compounds for two-phase produced OO, being the most evident. In brief, based on our results, the method used for the production of OO mainly affects its polar constituents and not the saponifiable part. The polar phenolics are more abundant in two-phase produced OO such as secoiridoids and phenylalcohols (Figure 9). Furthermore, the milling conditions such as time and temperature seems to affect considerably the composition of OO.

### UPLC-HRMS Analysis

**Figure 10:**
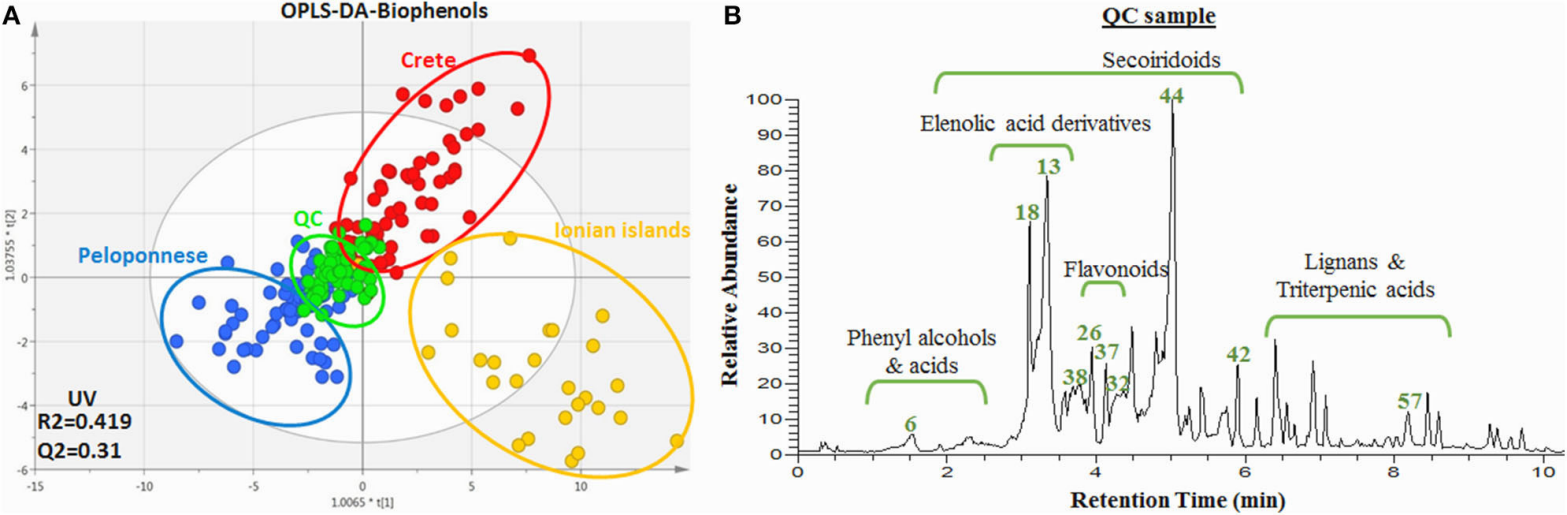
**(A)** OPLS-DA scores plot of the biophenol extract samples acquired via UPLC-HRMS. Plot includes the total number of observations in color scale according to geographical origin ClassID; Peloponnese (blue), Crete (red), Ionian islands (yellow). QC sample are also included (green). R2/Q2 model parameters are annotated (bottom-left). **(B)** Base peak (BP) chromatogram of the QC (pooled sample). Certain metabolites are annotated.

Except FIA-MRMS, LC-HRMS was also used for the analysis of samples as a more established method. In this case, only biophenol extracts were analyzed due to well-known restrictions in the analysis of intact oils with LC-MS techniques. A similar procedure as with FIA-MRMS was followed but also taking advantage of the separation factor in the LC. From 208 observations, 1,687 variables (*m/z*@Rt) were initially detected. Following the same work-flow as in FIA-MRMS, after data preprocessing, unsupervised and supervised methods were applied for all quality parameters. In Figure 10, the OPLS-DA scores plot is presented according to geographical origin (Table A3 in Supplementary Material).

It is worth mentioning that similar discrimination pattern is observed with the respective plot from FIA-MRMS analysis, nonetheless with higher dispersion. Samples from Crete and Ionian islands were grouped separately from Peloponnese samples in PC1 and between them in PC2. QC samples (pooled), as expected, were centralized with limited dispersion indicating appropriate analytical procedure and good data fitting (Figure A25 in Supplementary Material). It is worth noting that similar clustering trends in the separation of Ionian and Cretan samples with Peloponnese samples is observed in comparison to Figure 2 (FIA-MRMS analysis). Again Ionian samples are characterized by extensive dispersion and high number of outliers.

Close inspection of the two plots (Figure 2D and Figure 10) reveals that absence of the LC dimension in FIA-MRMS didn't affect the data derived. In contrast, the grouping is significantly improved with less dispersion and enhanced fitting parameters. From the calculated VIP values, 55 features were selected as statistically significant with VIP >1 and were forwarded for identification. It is worth notice that more statistically significant loadings were found with FIA–MRMS compared to LC-Orbitrap MS, as is shown from the corresponding VIP lists. Moreover, all significant features detected with LC-Orbitrap MS were detected as well as with FIA-MRMS (Table A2 in Supplementary Material). These results indicate that using FIA instead of LC doesn't affect neither the number nor the identity of the detected compounds and on the other hand allows the monitoring of compounds with variable physicochemical properties such as lipophilic (FAs, TGs, DGs) and hydrophilic (phenols, triterpenic acids) compounds at the same time.

As mentioned already, the LC-MS analysis was performed in the current study not only to verify the FIA-MRMS findings but also to be used in the dereplication work-flow and the identification of marker compounds and classes. Very critical for the identification procedure were the MS/MS spectra as well as the Rt. Moreover, the use of reference standards (level 1) enhanced further the identification confidence. Their use was focused on polar constituents of OO, which are less investigated compared to lipophilic compounds, and the availability of commercial reference standard is restricted. In Figure 10 some indicative markers unambiguously identified are annotated on the base peak profile of the pooled sample. Also, main chemical groups, e.g., phelylalcohols, secoiridoids, flavonoids, triterpenic acids, are designated. It is interesting to highlight for instance that following this process oleocanthalic acid, a structural isomer of oleacein and an oxidized form of oleocanthal, was identified. Compared to its isomer oleacein, which is one of the major biophenols in Greek EVOOs, it is eluted in a different Rt and shows a different fragmentation pattern in MS/MS while it's not commercially available (Figures A6, A7 in Supplementary Material). Oleocanthalic acid was recently found as a new constituent of OO (Figure 1) and so far hasn't been mentioned as a marker compound.

Overall, this comparison shows that FIA-MRMS doesn't fall behind to LC-Orbitrap MS in metabolic profiling approaches; in contrast, it offers better visualization and prediction models, much more significant features, and therefore marker compounds, while it diminishes drastically analysis time. Most importantly, using FIA-MRMS, the LC restrictions are avoided, allowing the detection of compound classes of different physicochemical properties expanding the applications capabilities. Additional parameters are also worth investigating in the future, and also this technique could be used with other matrixes aside from olive oil. Nonetheless, the MS/MS data remain a valuable tool in identification process, increasing the level of confidence. Even so, the use of reference standards might help considerably toward this direction even if it is not always feasible when dealing with food products and generally natural products.

## Conclusion

Edible oils are an integral part of a human's daily diet. A plethora of scientific evidence has underlined the significance of food and dietary-exposome to human health well-being and the prevention as well as protection from chronic ailments. Olive oil (OO), a predominant component of the Mediterranean diet, stands out among foods as superior oil with unique organoleptic properties as well as beneficial effects to health and most importantly protection against chronic diseases. However, due to its high added value, there are widespread cases of fraud with significant impact on the economy, especially of Europe and Mediterranean countries, as well as on consumers' health. However, ensuring authenticity and quality of OO is quite a multifaceted issue due to its complex, unique, and quite variable composition. At the heart of OO research in recent years are the polyphenols, which are characterized by numerous important biological properties. However, there is a lack of fast and reliable methods to assess the quality and authenticity of OO.

In the current study, FIA-MRMS was employed using an untargeted metabolic profiling approach, proposing a method for quality control of OO focusing in particular on Greek EVOOs. Moreover, LC-Orbitrap MS was integrated for comparison purposes. Specifically, EVOO monovarietal (Koroneiki) samples were thoroughly selected from different areas of Greece and specific parameters influencing OO composition, i.e., geographical region, cultivation practice, and production procedure were investigated. Special attention was given to metadata, qualifying the appropriate ones subjected to the study. Both biophenols after extraction and intact EVOOs were analyzed using FIA-MRMS and in parallel LC-Orbitrap were used for the analysis of polyphenols samples. Data derived from both techniques were subjected to MDA and correlated. Clear classification of EVOO samples was achieved in both types of samples with FIA-MRMS using for class discrimination all the parameters under investigation. Taking advantage of the excellent resolution and accuracy of MRMS, it was possible to identify specific compounds responsible for the observed classification with high confidence. Marker compounds and/or classes characterizing EVOOs from different geographical origin, produced from trees with different agronomical procedures and with different milling procedures, were proposed. Also, a minimum analysis time, i.e., 2 and 8 min, for intact EVOOs and biophenols, respectively, was required with almost no prior sample preparation. Furthermore, the use of FIA-MRMS enabled the simultaneous monitoring of both lipophilic compounds such as TGAs, DGAs, and FFA as well as polyphenols and in particular secoiridoids (e.g., elenolic acid, oleacein, oleocanthal, oleocanthalic acid, aldehydic form of oleuropein aglycon), phenylethanoids (e.g., tyrosol, hydroxytyrosol), lignans (e.g., acetoxypinoresinol, pinoresinol), flavonoids (e.g., luteolin, apigenin), plenolic acids (e.g., benzoic acid, protocatehuic acid) as well as triterpenic acids (e.g., oleanolic acid, maslinic acid).

Interestingly, better projection and prediction models, with increased number of statistically significant features, were generated from FIA-MRMS analysis compared to the more established LC-Orbitrap MS platform. Nevertheless, the use of the later technique when used as dereplication tool for known constituents offering HRMS/MS data assisted considerably in the identification confidence. Moreover, the use of numerous biophenols reference standards, many of them not commercially available, resulted in unambiguous identification of certain quality markers.

To our knowledge it is the first time that Koroneiki variety, the most abundant in Greece, is thoroughly investigated toward both glycerol part and phenols with a single method, and in correlation with two very important factors for EVOO quality, agronomical practice and production procedure. Moreover, certain markers but also compounds classes were suggested as quality and authenticity markers. Therefore, FIA-MRMS is proven to be a versatile, fast, and efficient method for analysis of OO comparable to other fast techniques such as NMR offering additionally increased sensitivity and identification confidence. It could be considered as a promising alternative for quality control purposes and authenticity assessment for a complex and highly variable matrix such as EVOO.

## Data Availability Statement

The raw data supporting the conclusions of this article will be made available by the authors, without undue reservation.

## Author Contributions

TN and MW participated in experiments performance and manuscript writing. PS and AB supervised special parts of the experimental procedure, while MH supervised the whole project and revised the final manuscript. All authors contributed to the article and approved the submitted version.

## Conflict of Interest

MW and AB are employed by the company Bruker Daltonik GmbH. The remaining authors declare that the research was conducted in the absence of any commercial or financial relationships that could be construed as a potential conflict of interest.

## Footnotes

^1^http://www.internationaloliveoil.org/estaticos/view/83-designations-and-definitions-of-olive-oils

^2^https://ec.europa.eu/jrc/en/research-topic/food-authenticity-and-qualit

## Abbreviations

OO: olive oil
FIA: flow injection analysis
MRMS: magnetic resonance mass spectrometry
EVOO: extra virgin olive oil
VOO: virgin olive oil
IOC: International Olive oil Council
PGI: protected geographical indication
PDO: protected designation of origin
MUFA: monounsaturated fatty acid
FA: fatty acids
EFSA: European Food Safety Authority
TGs: triacylglycerides
DGs: diglycerides
FFAs: free fatty acids
MUFA: monounsaturated fatty acids
MDA: multivariate data analysis
TPC: total phenolic content
NaTFA: sodium trifluoroacetate
MeOH: methanol
ESI: electrospray ionization source
PCA: principal component analysis
OPLS-DA: orthogonal partial least squares-discriminant analysis
VIP: variable importance in projection
UVS: unit variance scaling.
