# Supplementary material for: Olive Oil Quality and Authenticity Assessment Aspects Employing FIA-MRMS and LC-Orbitrap MS Metabolomic Approaches
Source: Front Public Health. 2020 Sep 25;8:558226. doi: 10.3389/fpubh.2020.558226 (PMC7545581; doi:10.3389/fpubh.2020.558226)
Supplement: Supplementary file 1 [file Data_Sheet_1.PDF]

Supplementary Material

Olive oil quality and authenticity assessment aspects employing FIA-MRMS and LC-Orbitrap MS metabolomic approaches.

Theodora Nikou[1], Matthias Witt[2], Panagiotis Stathopoulos[1], Aiko Barsch[2], Maria Halabalaki[1*]

[1]Department of Pharmacognosy and Natural Products Chemistry, Faculty of Pharmacy, National and Kapodistrian University of Athens, Athens, Greece

[2]Bruker Daltonik GmbH, Bremen, Germany

Table A1: Metadata of collected samples and TPC values of biophenols extracts via Folin-Ciocalteu assay.

Code	Origin	Variety	Type of cultivation	Production procedure	TPC (mg GAE Kg^{-1} EVOO)
**OLE410**	Heraklion	Koroneiki	Integrated	Three-phase	272
**OLE411**	Heraklion	Koroneiki	Integrated	Three-phase	380
**OLE412**	Lasithi	Koroneiki	Organic	Two-phase	375
**OLE413**	Lasithi	Koroneiki	Organic	Two-phase	317
**OLE416**	Heraklion	Koroneiki	Integrated	Three-phase	322
**OLE417**	Lasithi	Koroneiki	Integrated	Two-phase	172
**OLE418**	Heraklion	Koroneiki	Conventional	Three-phase	276
**OLE419**	Lasithi	Koroneiki	Organic	Two-phase	470
**OLE420**	Lasithi	Koroneiki	Organic	Two-phase	607
**OLE421**	Heraklion	Koroneiki	Organic	Three-phase	194
**OLE422**	Heraklion	Koroneiki	Organic	Three-phase	167
**OLE423**	Kefalonia	Koroneiki-Kefalonia	Conventional	Two-phase	190
**OLE424**	Kefalonia	Koroneiki-Kefalonia	Conventional	Two-phase	64
**OLE425**	Kefalonia	Koroneiki-Kefalonia	Conventional	Two-phase	160
**OLE426**	Kefalonia	Koroneiki-Kefalonia	Conventional	Two-phase	116
**OLE427**	Kefalonia	Koroneiki-Kefalonia	Conventional	Two-phase	194
**OLE428**	Kefalonia	Koroneiki-Kefalonia	Conventional	Two-phase	175

**OLE429**	Kefalonia	Koroneiki-Kefalonia	Conventional	Two-phase	126
**OLE430**	Kefalonia	Koroneiki-Kefalonia	Conventional	Two-phase	198
**OLE431**	Kefalonia	Koroneiki-Kefalonia	Conventional	Two-phase	275
**OLE432**	Kefalonia	Koroneiki-Kefalonia	Conventional	Two-phase	272
**OLE433**	Kefalonia	Koroneiki-Kefalonia	Conventional	Two-phase	146
**OLE434**	Kefalonia	Koroneiki-Kefalonia	Conventional	Two-phase	277
**OLE435**	Kefalonia	Koroneiki-Kefalonia	Conventional	Two-phase	216
**OLE436**	Kefalonia	Koroneiki-Kefalonia	Conventional	Two-phase	141
**OLE437**	Kefalonia	Koroneiki-Kefalonia	Conventional	Two-phase	266
**OLE438**	Kefalonia	Koroneiki-Kefalonia	Conventional	Two-phase	82
**OLE439**	Kefalonia	Koroneiki-Kefalonia	Conventional	Two-phase	129
**OLE440**	Kefalonia	Koroneiki-Kefalonia	Conventional	Two-phase	169
**OLE441**	Ithaki	Koroneiki- Thiaki	Organic	Two-phase	239
**OLE442**	Ithaki	Koroneiki- Thiaki	Organic	Two-phase	172
**OLE443**	Ithaki	Koroneiki- Thiaki	Organic	Two-phase	259
**OLE444**	Ithaki	Koroneiki- Thiaki	Conventional	Two-phase	199
**OLE445**	Ithaki	Koroneiki- Thiaki	Organic	Two-phase	276
**OLE446**	Ithaki	Koroneiki- Thiaki	Organic	Two-phase	88
**OLE447**	Ithaki	Koroneiki- Thiaki	Organic	Two-phase	191
**OLE448**	Ithaki	Koroneiki- Thiaki	Organic	Two-phase	224
**OLE449**	Ithaki	Koroneiki- Thiaki	Organic	Two-phase	139
**OLE450**	Ithaki	Koroneiki- Thiaki	Organic	Two-phase	291
**OLE451**	Lakonia	Koroneiki	Organic	Three-phase	200
**OLE452**	Lakonia	Koroneiki	Organic	Three-phase	146
**OLE453**	Lakonia	Koroneiki	Conventional	Three-phase	133
**OLE454**	Lakonia	Koroneiki	Conventional	Three-phase	88
**OLE455**	Lakonia	Koroneiki	Conventional	Three-phase	73
**OLE456**	Lakonia	Koroneiki	Conventional	Three-phase	107
**OLE457**	Lakonia	Koroneiki	Conventional	Three-phase	167
**OLE458**	Lakonia	Koroneiki	Conventional	Three-phase	111
**OLE459**	Lakonia	Koroneiki	Conventional	Three-phase	154
**OLE460**	Lakonia	Koroneiki	Conventional	Three-phase	137
**OLE464**	Heraklion	Koroneiki	Integrated	Three-phase	141
**OLE465**	Lasithi	Koroneiki	Integrated	Three-phase	263
**OLE466**	Lasithi	Koroneiki	Integrated	Three-phase	239
**OLE467**	Lasithi	Koroneiki	Organic	Two-phase	272

**OLE468**	Lasithi	Koroneiki	Integrated	Two-phase	144
**OLE469**	Lasithi	Koroneiki	Integrated	Two-phase	190
**OLE470**	Lasithi	Koroneiki	Organic	Two-phase	239
**OLE471**	Lasithi	Koroneiki	Integrated	Three-phase	204
**OLE472**	Lasithi	Koroneiki	Integrated	Two-phase	350
**OLE473**	Lasithi	Koroneiki	Integrated	Two-phase	186
**OLE474**	Lasithi	Koroneiki	Integrated	Two-phase	170
**OLE475**	Lasithi	Koroneiki	Integrated	Two-phase	163
**OLE476**	Heraklion	Koroneiki	Integrated	Two-phase	174
**OLE477**	Lasithi	Koroneiki	Integrated	Three-phase	197
**OLE478**	Lasithi	Koroneiki	Integrated	Three-phase	174
**OLE479**	Lasithi	Koroneiki	Integrated	Three-phase	141
**OLE480**	Heraklion	Koroneiki	Integrated	Three-phase	113
**OLE487**	Messinia	Koroneiki	Conventional	Two-phase	243
**OLE488**	Messinia	Koroneiki	Conventional	Two-phase	227
**OLE489**	Messinia	Koroneiki	Organic	Two-phase	178
**OLE490**	Messinia	Koroneiki	Organic	Two-phase	172
**OLE491**	Messinia	Koroneiki	Conventional	Two-phase	234
**OLE492**	Messinia	Koroneiki	Conventional	Three-phase	168
**OLE493**	Messinia	Koroneiki	Conventional	Three-phase	186
**OLE494**	Messinia	Koroneiki	Conventional	Two-phase	308
**OLE495**	Messinia	Koroneiki	Organic	Two-phase	115
**OLE496**	Messinia	Koroneiki	Conventional	Three-phase	170
**OLE497**	Messinia	Koroneiki	Conventional	Three-phase	140
**OLE498**	Messinia	Koroneiki	Organic	Two-phase	231
**OLE499**	Messinia	Koroneiki	Conventional	Two-phase	164
**OLE500**	Messinia	Koroneiki	Conventional	Two-phase	192
**OLE501**	Messinia	Koroneiki	Conventional	Two-phase	175
**OLE502**	Messinia	Koroneiki	Organic	Two-phase	203
**OLE503**	Messinia	Koroneiki	Conventional	Two-phase	267
**OLE504**	Messinia	Koroneiki	Conventional	Two-phase	113
**OLE505**	Messinia	Koroneiki	Conventional	Two-phase	198
**OLE506**	Messinia	Koroneiki	Conventional	Two-phase	200
**OLE509**	Heraklion	Koroneiki	Conventional	Three-phase	120
**OLE510**	Heraklion	Koroneiki	Conventional	Three-phase	216
**OLE511**	Lasithi	Koroneiki	Integrated	Three-phase	185
**OLE512**	Lasithi	Koroneiki	Integrated	Three-phase	76

**OLE513**	Lasithi	Koroneiki	Conventional	Two-phase	254
**OLE514**	Lasithi	Koroneiki	Conventional	Three-phase	144
**OLE515**	Lasithi	Koroneiki	Organic	Two-phase	372
**OLE516**	Heraklion	Koroneiki	Organic	Two-phase	280
**OLE517**	Lasithi	Koroneiki	Conventional	Three-phase	208
**OLE518**	Lasithi	Koroneiki	Conventional	Two-phase	130
**OLE519**	Lasithi	Koroneiki	Conventional	Two-phase	185
**OLE520**	Lasithi	Koroneiki	Conventional	Two-phase	192
**OLE521**	Messinia	Koroneiki	Conventional	Traditional	217
**OLE522**	Messinia	Koroneiki	Conventional	Traditional	111
**OLE523**	Messinia	Koroneiki	Conventional	Traditional	274
**OLE524**	Messinia	Koroneiki	Conventional	Traditional	295
**OLE534**	Lasithi	Koroneiki	Conventional	Two-phase	220
**OLE535**	Lasithi	Koroneiki	Organic	Two-phase	273
**OLE536**	Lasithi	Koroneiki	Conventional	Three-phase	198
**OLE543**	Messinia	Koroneiki	Conventional	Three-phase	125
**OLE544**	Heraklion	Koroneiki	Conventional	Three-phase	229
**OLE545**	Lasithi	Koroneiki	Conventional	Two-phase	357
**OLE546**	Lasithi	Koroneiki	Conventional	Three-phase	588
**OLE547**	Lasithi	Koroneiki	Organic-non certified	Two-phase	312
**OLE548**	Lasithi	Koroneiki	Conventional	Two-phase	323
**OLE549**	Lasithi	Koroneiki	Organic	Three-phase	199
**OLE550**	Lasithi	Koroneiki	Organic	Two-phase	221
**OLE555**	Messinia	Koroneiki	Conventional	Three-phase	224
**OLE556**	Messinia	Koroneiki	Conventional	Three-phase	192
**OLE557**	Lakonia	Koroneiki	Organic	Three-phase	198
**OLE558**	Lakonia	Koroneiki	Organic	Three-phase	172
**OLE559**	Lakonia	Koroneiki	Organic	Three-phase	131
**OLE560**	Lakonia	Koroneiki	Organic	Three-phase	119
**OLE561**	Lakonia	Koroneiki	Organic	Three-phase	186
**OLE569**	Lakonia	Koroneiki	Organic-non certified	Two-phase	174
**OLE570**	Lakonia	Koroneiki	Organic-non certified	Two-phase	133
**OLE571**	Lakonia	Koroneiki	Organic-non certified	Two-phase	216
**OLE572**	Lakonia	Koroneiki	Organic-non certified	Two-phase	136
**OLE573**	Lakonia	Koroneiki	Organic-non certified	Two-phase	112
**OLE574**	Lakonia	Koroneiki	Organic-non certified	Two-phase	180

**OLE575**	Lakonia	Koroneiki	Organic-non certified	Two-phase	136
**OLE576**	Lakonia	Koroneiki	Organic-non certified	Two-phase	172
**OLE577**	Lakonia	Koroneiki	Organic-non certified	Two-phase	95
**OLE578**	Lakonia	Koroneiki	Organic-non certified	Two-phase	97
**OLE579**	Lakonia	Koroneiki	Organic-non certified	Two-phase	242
**OLE580**	Lakonia	Koroneiki	Organic-non certified	Two-phase	197
**OLE581**	Lakonia	Koroneiki	Organic-non certified	Two-phase	202
**OLE582**	Lakonia	Koroneiki	Organic-non certified	Two-phase	189
**OLE589**	Heraklion	Koroneiki	Organic	Three-phase	138
**OLE590**	Heraklion	Koroneiki	Organic	Three-phase	191
**OLE591**	Heraklion	Koroneiki	Organic	Three-phase	189
**OLE592**	Heraklion	Koroneiki	Organic	Three-phase	271
**OLE593**	Heraklion	Koroneiki	Conventional	Three-phase	94
**OLE594**	Heraklion	Koroneiki	Conventional	Three-phase	217
**OLE595**	Heraklion	Koroneiki	Organic	Three-phase	156
**OLE596**	Heraklion	Koroneiki	Organic	Three-phase	132
**OLE597**	Heraklion	Koroneiki	Conventional	Three-phase	219
**OLE598**	Heraklion	Koroneiki	Conventional	Three-phase	212
**OLE599**	Heraklion	Koroneiki	Conventional	Three-phase	168
**OLE600**	Heraklion	Koroneiki	Conventional	Three-phase	203
**OLE601**	Heraklion	Koroneiki	Conventional	Three-phase	240
**OLE602**	Heraklion	Koroneiki	Conventional	Three-phase	207
**OLE607**	Heraklion	Koroneiki	Conventional	Three-phase	145
**OLE608**	Heraklion	Koroneiki	Conventional	Three-phase	233
**OLE609**	Heraklion	Koroneiki	Conventional	Three-phase	237
**OLE612**	Heraklion	Koroneiki	Integrated	Three-phase	227
**OLE613**	Heraklion	Koroneiki	Conventional	Three-phase	223
**OLE614**	Heraklion	Koroneiki	Conventional	Three-phase	145
**OLE615**	Heraklion	Koroneiki	Conventional	Three-phase	147
**OLE616**	Heraklion	Koroneiki	Conventional	Three-phase	114
**OLE617**	Heraklion	Koroneiki	Conventional	Three-phase	111
**OLE618**	Heraklion	Koroneiki	Conventional	Three-phase	249
**OLE635**	Kefalonia	Koroneiki-Kefalonia	Organic-non certified	Two-phase	230
**OLE636**	Kefalonia	Koroneiki-Kefalonia	Organic-non certified	Two-phase	98
**OLE637**	Kefalonia	Koroneiki-Kefalonia	Organic-non certified	Two-phase	239
**OLE638**	Kefalonia	Koroneiki-Kefalonia	Organic-non certified	Two-phase	132
**OLE639**	Kefalonia	Koroneiki-Kefalonia	Organic-non certified	Two-phase	113

**OLE640**	Kefalonia	Koroneiki-Kefalonia	Organic-non certified	Two-phase	214
**OLE647**	Heraklion	Koroneiki	Conventional	Three-phase	196
**OLE648**	Heraklion	Koroneiki	Conventional	Three-phase	133
**OLE649**	Heraklion	Koroneiki	Conventional	Three-phase	133
**OLE650**	Heraklion	Koroneiki	Conventional	Three-phase	159
**OLE651**	Heraklion	Koroneiki	Conventional	Three-phase	141
**OLE652**	Heraklion	Koroneiki	Conventional	Two-phase	164
**OLE653**	Heraklion	Koroneiki	Conventional	Three-phase	269
**OLE654**	Heraklion	Koroneiki	Conventional	Three-phase	250
**OLE655**	Heraklion	Koroneiki	Conventional	Three-phase	233
**OLE656**	Lasithi	Koroneiki	Integrated	Three-phase	269
**OLE657**	Heraklion	Koroneiki	Conventional	Three-phase	120
**OLE677**	Lakonia	Koroneiki	Integrated	Two-phase	218
**OLE678**	Lakonia	Koroneiki	Conventional	Two-phase	180
**OLE679**	Lakonia	Koroneiki	Integrated	Two-phase	191
**OLE680**	Lakonia	Koroneiki	Integrated	Two-phase	251
**OLE681**	Lakonia	Koroneiki	Integrated	Two-phase	172
**OLE682**	Lakonia	Koroneiki	Integrated	Two-phase	196
**OLE683**	Lakonia	Koroneiki	Integrated	Two-phase	368
**OLE684**	Lakonia	Koroneiki	Integrated	Two-phase	306
**OLE685**	Lakonia	Koroneiki	Integrated	Two-phase	367
**OLE687**	Lakonia	Koroneiki	Integrated	Two-phase	146
**OLE688**	Lakonia	Koroneiki	Integrated	Two-phase	165
**OLE689**	Lakonia	Koroneiki	Conventional	Two-phase	314
**OLE690**	Lakonia	Koroneiki	Integrated	Two-phase	197
**OLE691**	Lakonia	Koroneiki	Conventional	Two-phase	120
**OLE692**	Lakonia	Koroneiki	Conventional	Two-phase	146
**OLE693**	Lakonia	Koroneiki	Integrated	Two-phase	173
**OLE694**	Lakonia	Koroneiki	Integrated	Two-phase	179
**OLE695**	Lakonia	Koroneiki	Integrated	Two-phase	281
**OLE696**	Lakonia	Koroneiki	Organic	Two-phase	220
**OLE697**	Lakonia	Koroneiki	Conventional	Two-phase	209
**OLE699**	Lakonia	Koroneiki	Conventional	Two-phase	198
**OLE700**	Lakonia	Koroneiki	Integrated	Two-phase	106
**OLE702**	Messinia	Koroneiki	Organic	Two-phase	247
**OLE703**	Messinia	Koroneiki	Organic	Two-phase	181

**OLE704**	Messinia	Koroneiki	Organic	Two-phase	63
**OLE705**	Messinia	Koroneiki	Organic	Two-phase	161
**OLE706**	Messinia	Koroneiki	Conventional	Three-phase	124
**OLE707**	Messinia	Koroneiki	Conventional	Three-phase	148
**OLE708**	Messinia	Koroneiki	Conventional	Three-phase	134
**OLE709**	Messinia	Koroneiki	Conventional	Three-phase	235
**OLE710**	Messinia	Koroneiki	Conventional	Three-phase	127
**OLE711**	Messinia	Koroneiki	Conventional	Three-phase	112
**OLE712**	Messinia	Koroneiki	Conventional	Three-phase	179

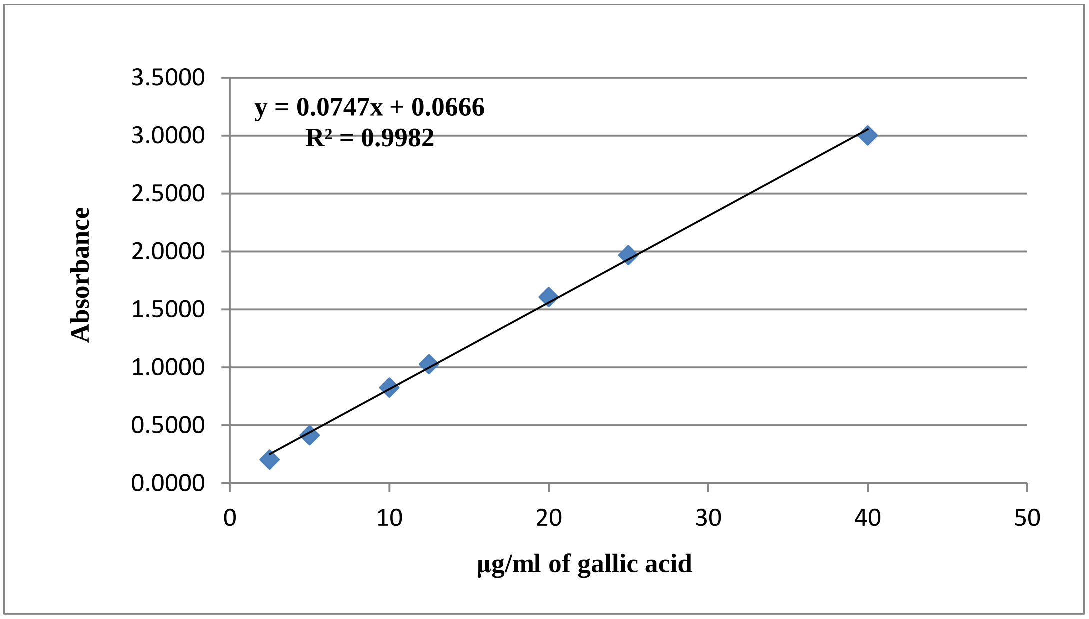

Figure A1: Standard calibration curve of gallic acid. The horizontal scale represents the used concentrations of gallic acid in µg/ml and the vertical scale the corresponding absorbance**.**

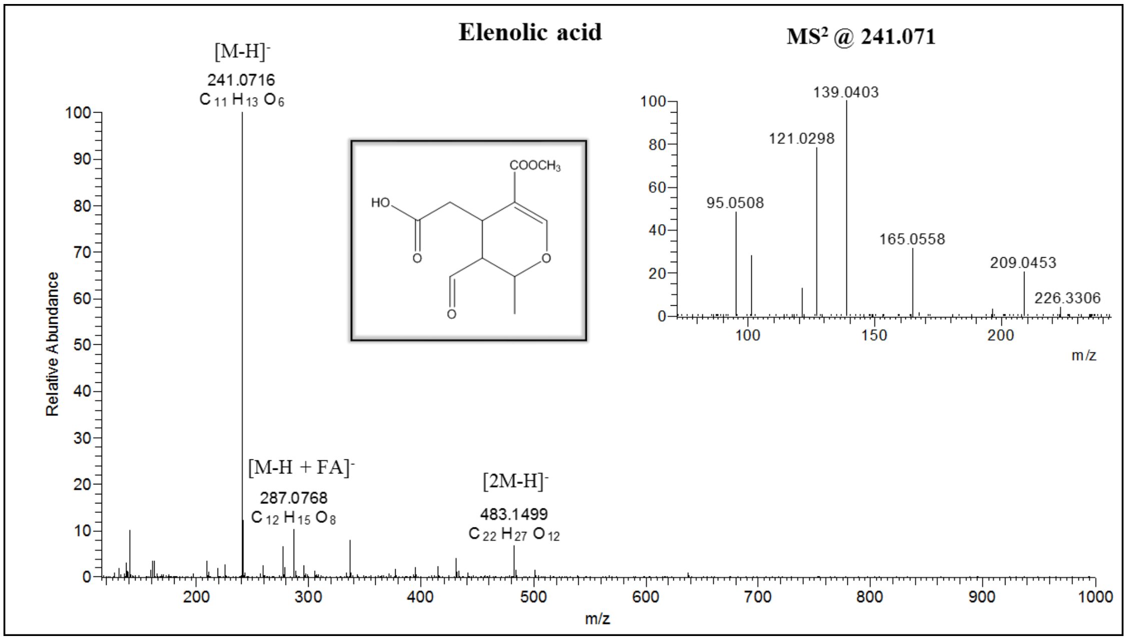

Figure A2: HRMS and HRMS/MS spectra of **elenolic acid (13)** in negative ion mode. Characteristic ions (adducts) are annotated.

.

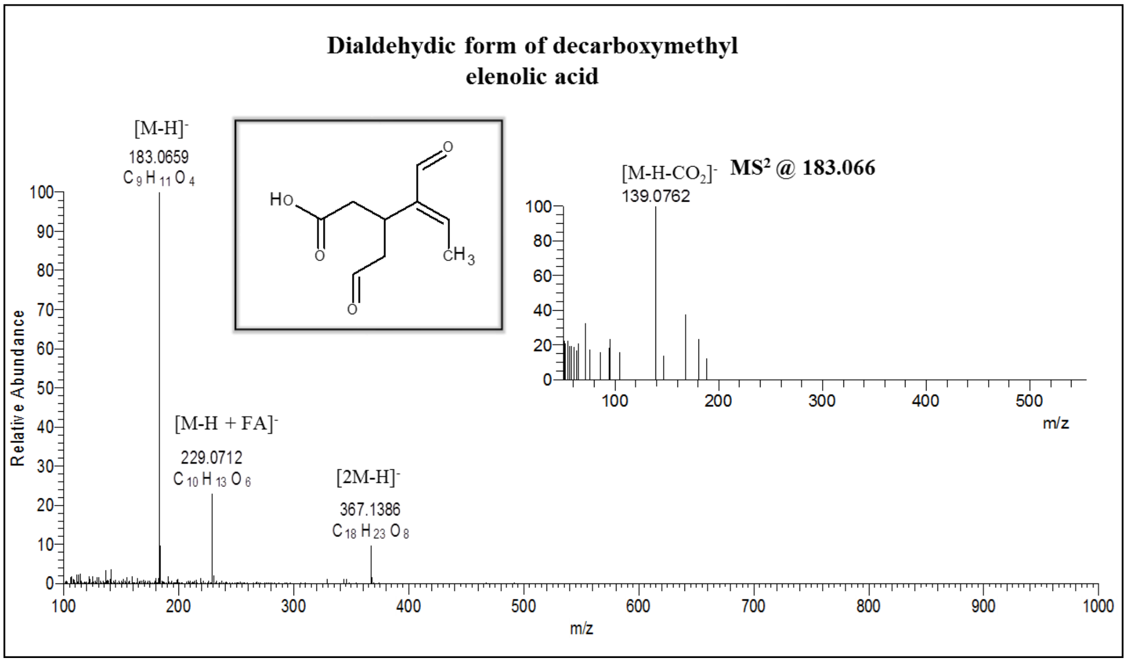

Figure A3: HRMS and HRMS/MS spectra of dialdehydic form of **decarboxymethyl elenolic acid (8)** in negative ion mode. Characteristic ions (adducts) are annotated.

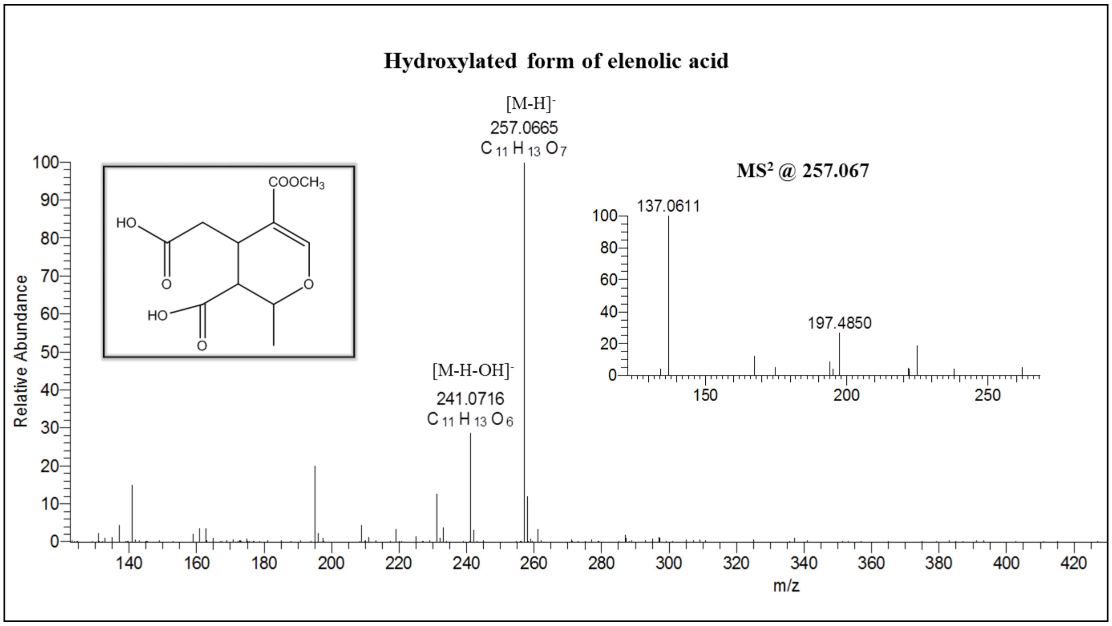

Figure A4: HRMS and HRMS/MS spectra of **hydroxylated form of elenolic acid (18)** in negative ion mode. Characteristic ions are annotated.

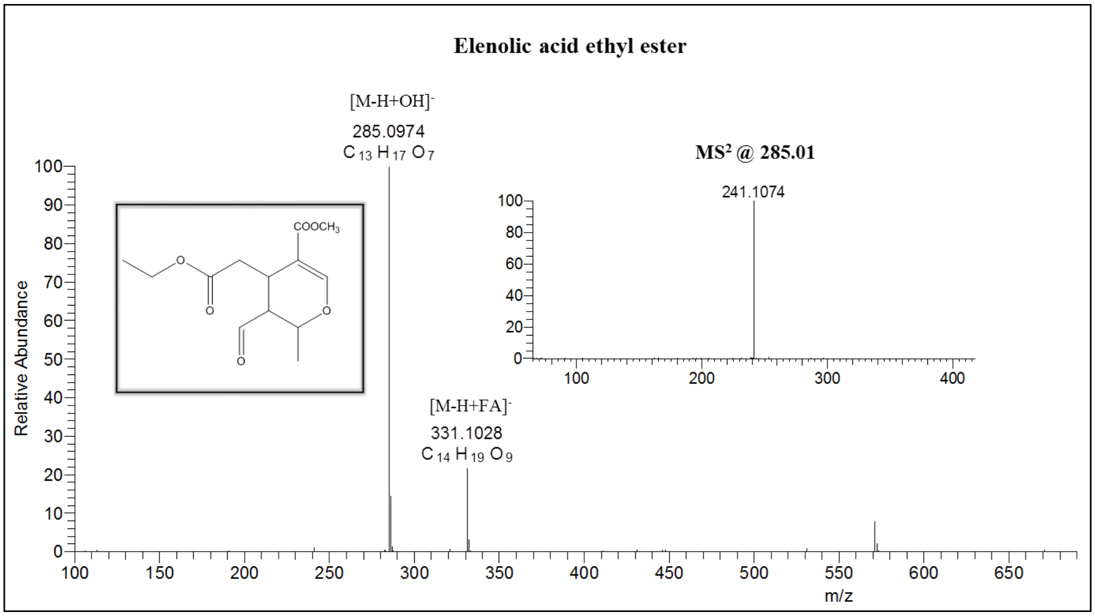

Figure A5: HRMS and HRMS/MS spectra of **elenolic acid ethyl ester (21)** in negative ion mode. Characteristic ions are annotated.

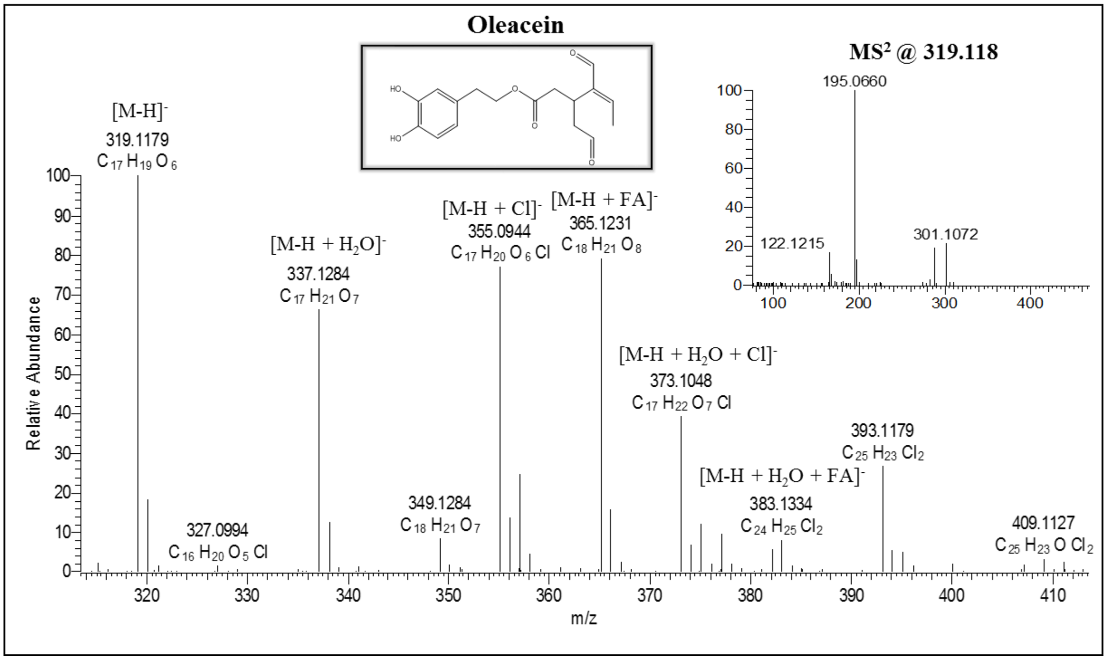

Figure A6: HRMS and HRMS/MS spectra of **oleacein (38)** in negative ion mode. Characteristic ions are annotated.

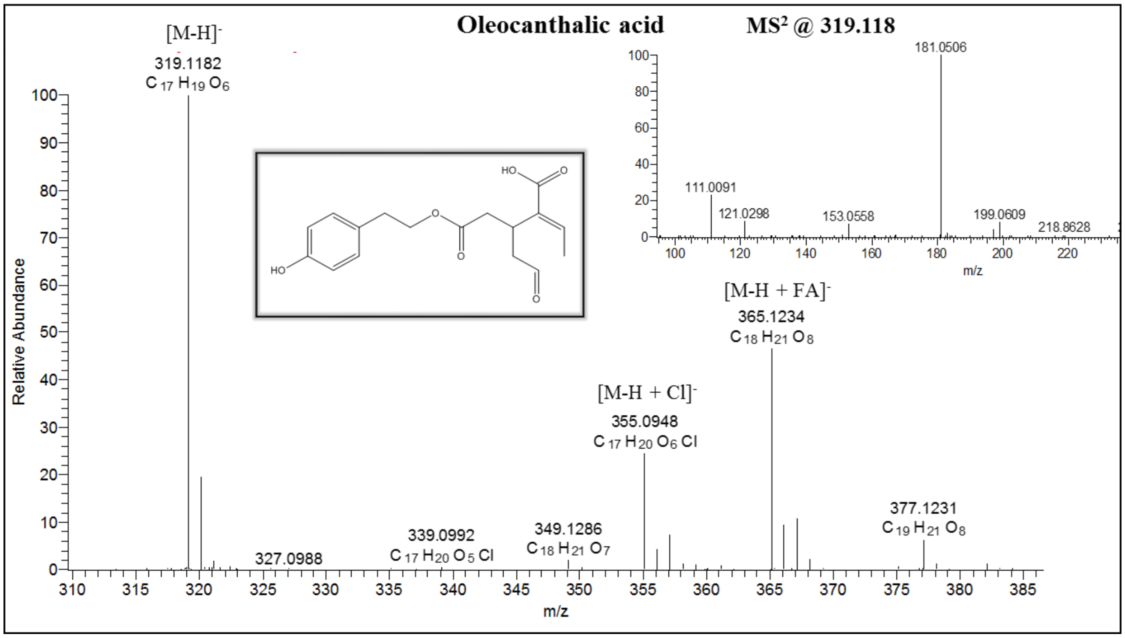

Figure A7: HRMS and HRMS/MS spectra of **oleocanthalic acid (37)** in negative ion mode. Characteristic adducts are annotated.

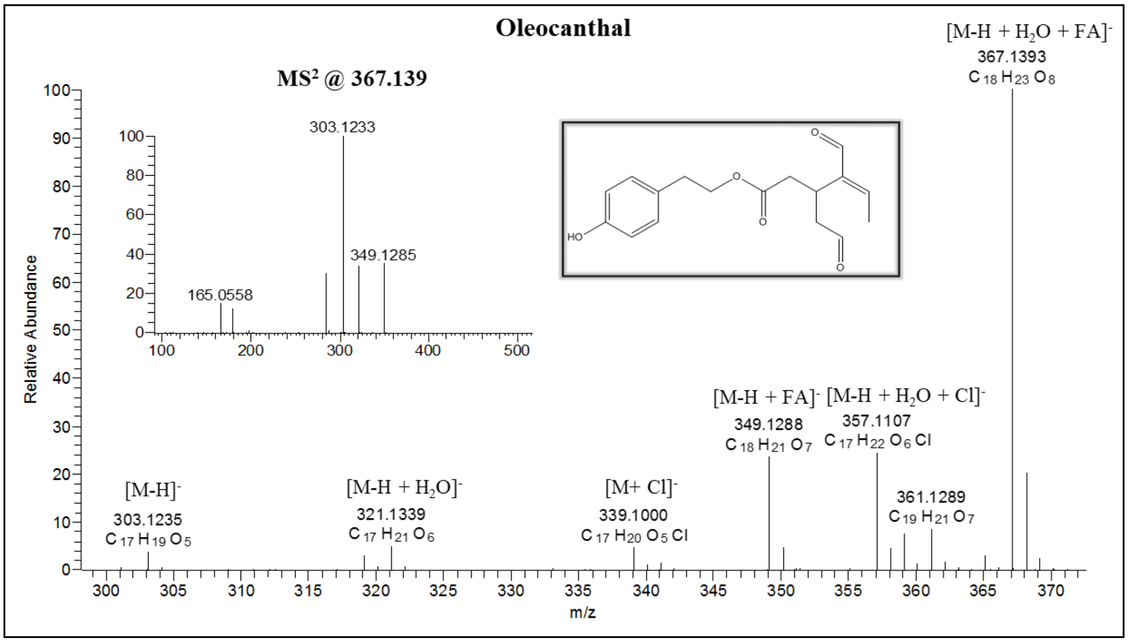

Figure A8: HRMS and HRMS/MS spectra of **oleocanthal (32)** in negative ion mode. Characteristic ions are annotated.

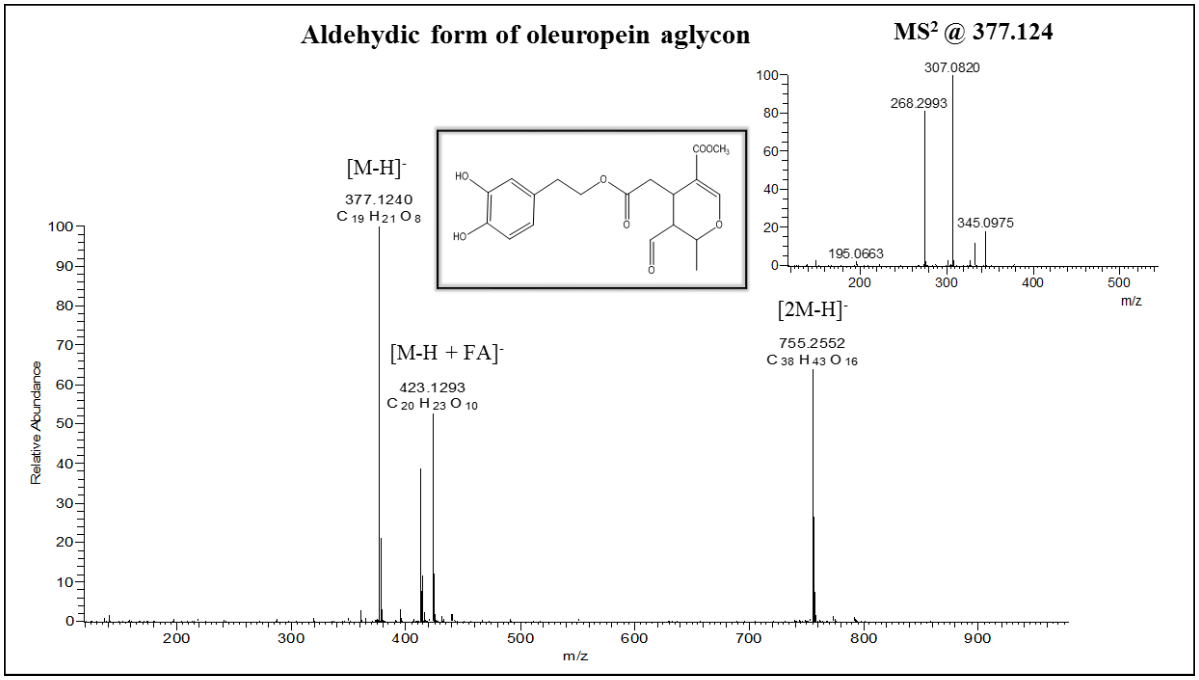

Figure A9: HRMS and HRMS/MS spectra of aldehydic form of **oleuropein aglycon (44)** in negative ion mode. Characteristic adducts are annotated.

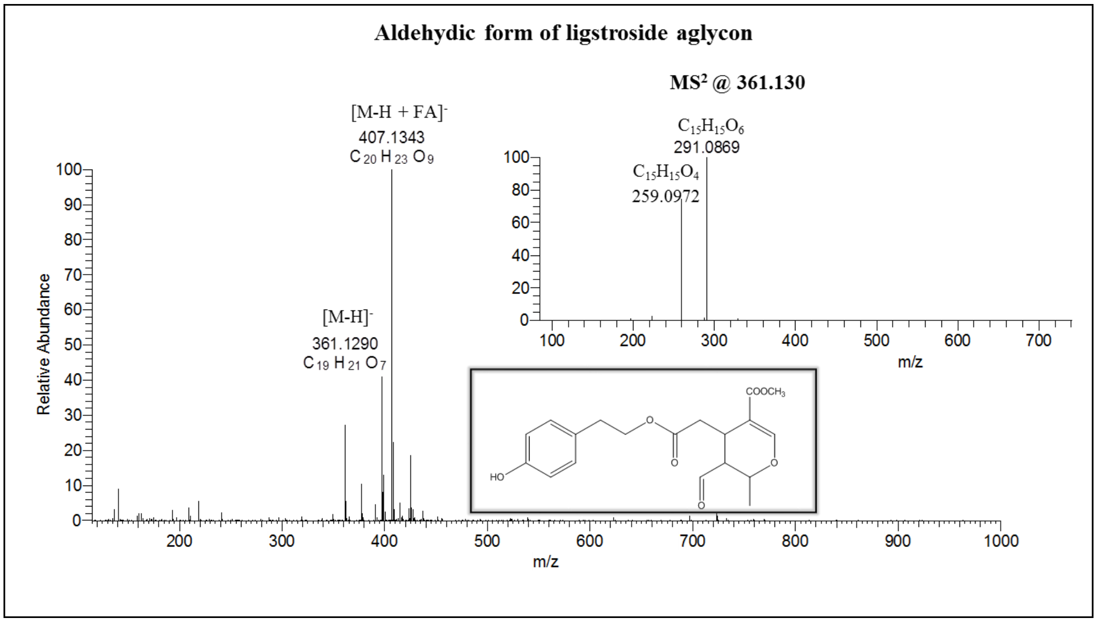

Figure A10: HRMS and HRMS/MS spectra of aldehydic form of **ligstroside aglycon (42)** in negative ion mode. Characteristic ions are annotated.

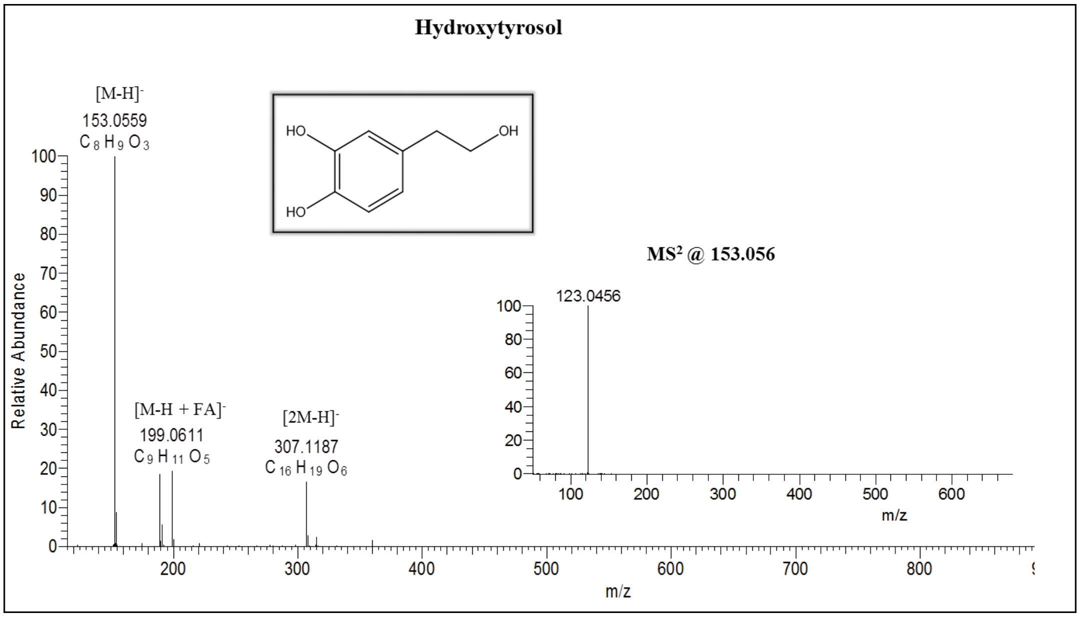

Figure A11: HRMS and HRMS/MS spectra of **hydroxytyrosol (6)** in negative ion mode. Characteristic adducts are annotated.

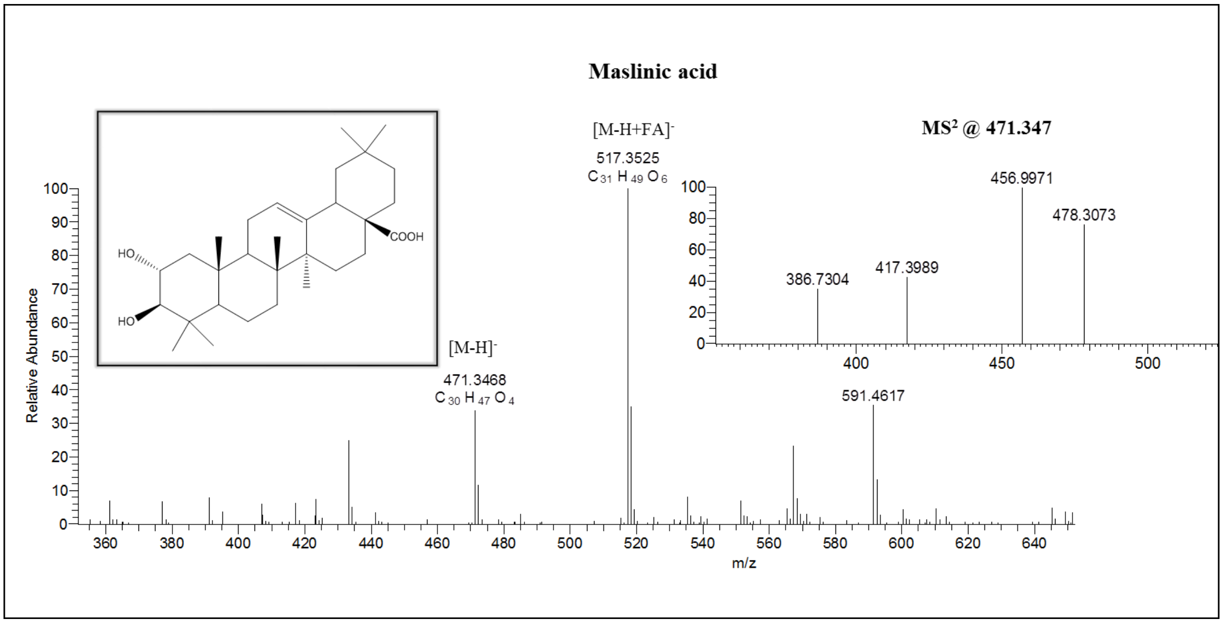

Figure A12: HRMS and HRMS/MS spectra of **maslinic acid (57)** in negative ion mode. Characteristic adducts are annotated.

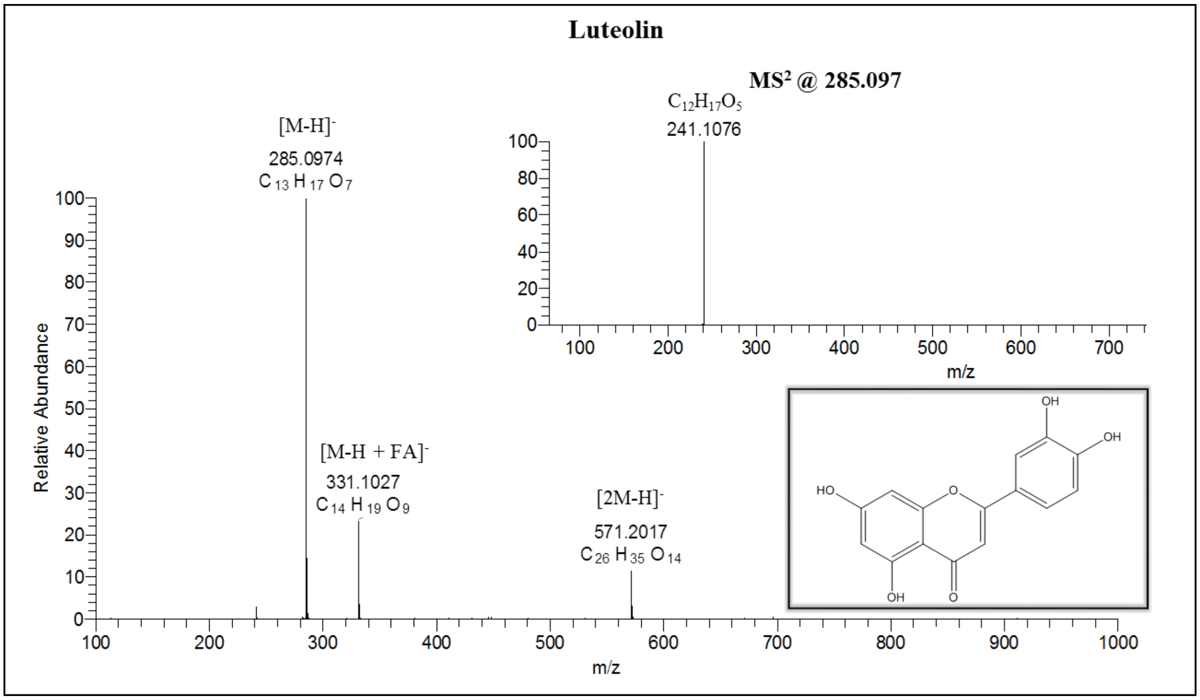

Figure A13: HRMS and HRMS/MS spectra of **luteolin (26)** in negative ion mode. Characteristic adducts are annotated.

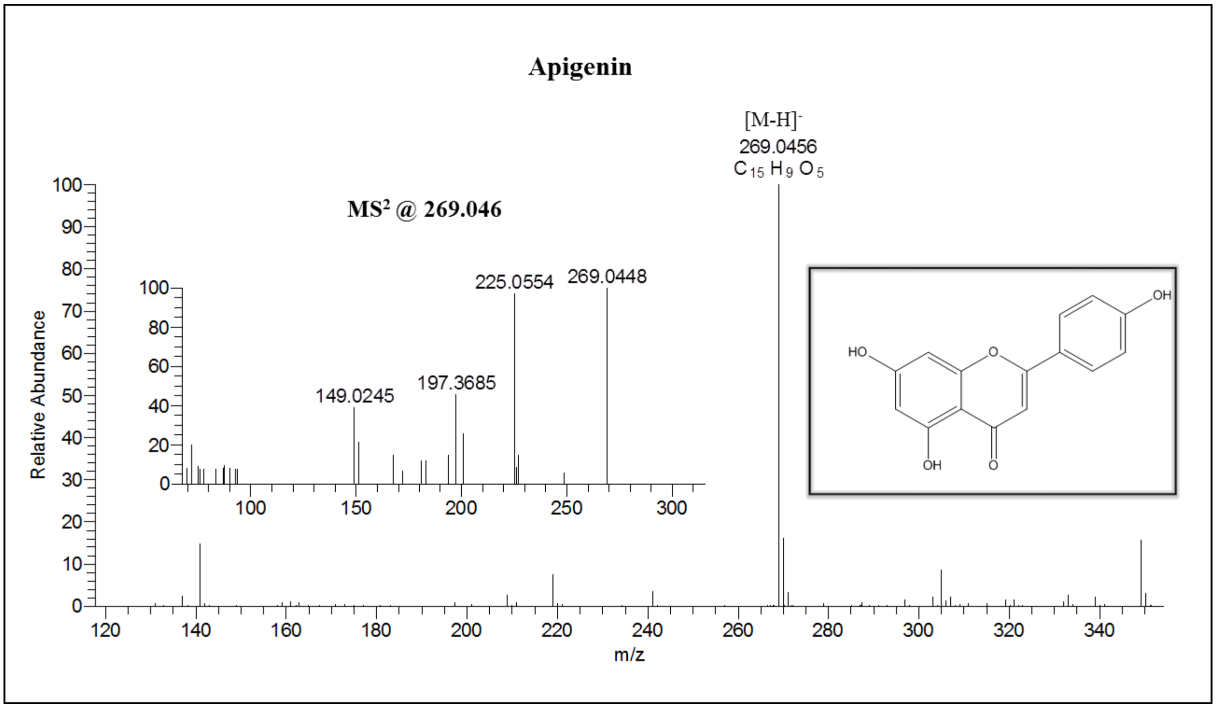

Figure A14*:* HRMS and HRMS/MS spectra of **apigenin (20)** in negative ion mode.

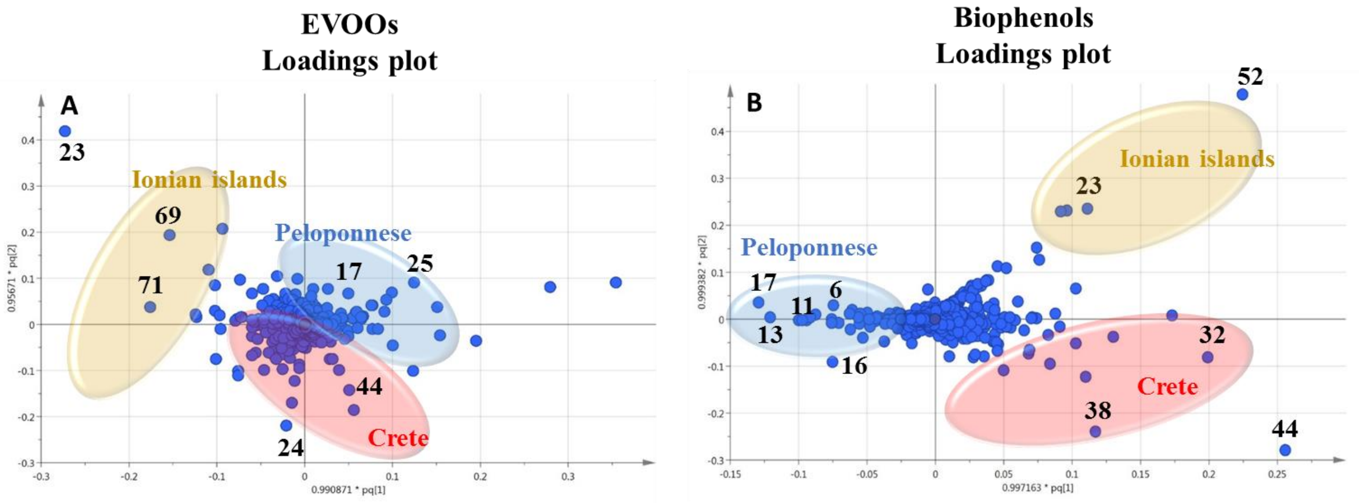

Figure A15: OPLS-DA loadings plot for geographical origin identification generated by FIA-MRMS for intact EVOOs (A) and biophenols (B) dataset. Representative statistically significant loadings corresponding to certain marker compounds are annotated in areas of Peloponnese (blue), Crete (red) and Ionian islands (yellow).

Table A2: Identified compounds with FIA-MRMS and UPLC-Orbitrap-MS. The identified compounds are numbered according to the first column. VIP scores are presented for each metabolite according to the studied Y-variable; origin, production procedure and cultivation practice. Mass error and mSigma values are generated by FIA-MRMS.

No	Experimental *m/z*	Molecular formula	Mass error [ppm]	mSigma	Compound name	VIP origin	VIP production procedure	VIP cultivation Practice	FIA-MRMS	LC-Orbitrap MS
1	119.034969	$C_4H_8O_4$	0.057	5.1	2-(2-hydroxyethoxy)acetic acid	5.22134	5.9023	5.92726	+	-
2	121.029503	$C_7H_6O_2$	0.071	∞	Benzoic acid	1.01405	-	1.09674	+	+
3	137.06081	$C_8H_{10}O_2$	0.077	1.9	Tyrosol	2.83547	3.1343	3.0046	+	+
4	143.107736	$C_8H_{16}O_2$	0.073	2.4	Octanoic acid	2.47795	2.86397	2.90721	+	+
5	153.01931	$C_7H_6O_4$	-0.002	4.8	Protocatehuic acid	4.19864	4.70888	4.8107	+	+
6	153.055719	$C_8H_{10}O_3$	0.031	2.4	Hydroxytyrosol	1.85303	2.10387	2.56499	+	+
7	179.071361	$C_{10}H_{12}O_3$	-0.061	5.3	Tyrosol Acetate	1.46927	1.4958	1.46246	+	+
8	183.066297	$C_9H_{12}O_4$	0.025	2.7	Dialdehydic form of decarboxymethyl Elenolic acid	1.94329	1.86022	1.93357	+	+
9	195.066278	$C_{10}H_{12}O_4$	-0.066	6.7	Hydroxytyrosol Acetate	5.84606	4.50373	4.5868	+	+

10	199.170347	$C_{12}H_{24}O_2$	-0.003	∞	Lauric acid	1.17209	1.24399	1.3038	+	-
11	225.076859	$C_{11}H_{14}O_5$	0.135	9.9	desoxy elenolic acid derivative	3.31405	1.57051	2.06128	+	+
12	227.201648	$C_{14}H_{28}O_2$	-0.025	6.5	Myristic acid	2.08587	2.20252	2.35945	+	-
13	241.071755	$C_{11}H_{14}O_6$	-0.072	6.1	Elenolic acid	6.52685	7.49337	7.9409	+	+
14	241.21729	$C_{15}H_{30}O_2$	-0.04	14.7	Pentadecanoic acid	1.628	1.76714	1.89093	+	-
15	253.217315	$C_{16}H_{30}O_2$	0.353	10.8	Palmitoleic acid	1.38489	1.27238	1.37402	+	-
16	255.087423	$C_{12}H_{16}O_6$	-0.104	8	Elenolic acid methyl ester	5.92739	5.10004	6.97496	+	+
17	255.232958	$C_{16}H_{32}O_2$	0.033	16.7	Palmitic acid	6.95458	5.94974	7.97047	+	-
18	257.066694	$C_{11}H_{14}O_7$	0.084	9.5	Hydroxylated form of elenolic acid	1.39886	1.73966	2.1057	+	+
19	267.23293	$C_{17}H_{32}O_2$	-0.120	∞	Margaroleic acid	1.03012	1.21267	1.19739	+	-
20	269.045551	$C_{15}H_{10}O_5$	0.009	11	Apigenin	2.17441	2.99418	2.66789	+	+
21	269.103081	$C_{13}H_{18}O_6$	0.213	∞	Elenolic acid ethyl ester	1.21439.	-	1.18804	+	+

22	269.248578	$C_{17}H_{34}O_2$	-0.098	9.2	Margaric acid	1.30501	1.35513	1.50447	+	-
23	279.23298	$C_{18}H_{32}O_2$	0.101	24.5	Linoleic acid	3.79235	1.50918	1.67573	+	-
24	281.248634	$C_{18}H_{34}O_2$	-0.095	5.7	Oleic acid	7.96697	3.60615	3.56477	+	-
25	283.264257	$C_{18}H_{36}O_2$	0.035	8.8	Stearic acid	3.94595	2.47538	4.2481	+	-
26	285.040502	$C_{15}H_{10}O_6$	0.166	16	Luteolin	2.55389	4.31307	1.81787	+	+
27	295.227889	$C_{18}H_{32}O_3$	-0.159	2.7	Hydroxylinoleic acid	9.94902	1.50918	1.99172	+	-
28	297.243504	$C_{18}H_{34}O_3$	-0.016	1.9	Hydroxyoleic acid	15.6066	11.8227	8.54964	+	-
29	299.056138	$C_{16}H_{12}O_6$	0.141	8.3	Luteolin-7 methyl-ether	1.59344	2.61407	2.29155	+	+
30	299.20161	$C_{20}H_{28}O_2$	-0.067	13.8	PUFA, C-20	3.11107	3.35868	3.53252	+	-
31	299.259173	$C_{18}H_{36}O_3$	-0.061	8.5	Hydroxystearic acid	3.3611	4.39046	3.35659	+	-
32	303.123779	$C_{17}H_{20}O_5$	-0.169	5.8	Oleocanthal	7.10945	6.12967	7.56752	+	+
33	303.232928	$C_{20}H_{32}O_2$	-0.083	∞	Arachidonic acid	-	-	1.05502	+	-
34	309.284317	$C_{20}H_{38}O_2$	-0.031	∞	Gondoic acid	1.26752	1.51175	-	+	-
35	311.295541	$C_{20}H_{40}O_2$	0.024	∞	Arachidic acid	1.82052	1.88345	1.12426	+	-

36	313.238436	$C_{18}H_{34}O_4$	-0.158	13.6	Octadecanedioic acid	3.99715	3.46563	2.50328	+	-
37	319.116536	$C_{17}H_{20}O_6$	-0.03	3.1	Oleocanthalic acid	2.05932	1.30741	-	-	+
38	319.118731	$C_{17}H_{20}O_6$	-0.064	3.7	Oleacein	8.37747	5.16096	6.61328	+	+
39	335.113652	$C_{17}H_{20}O_7$	0.091	18.7	Hydroxylated derivative of decarboxymethyl oleuropein aglycon	2.66469	1.72474	2.23065	+	+
40	339.232889	$C_{23}H_{32}O_2$	-0.108	41	Docosanoic acid	5.41017	6.1416	6.20101	+	-
41	357.134425	$C_{20}H_{22}O_6$	0.172	12.8	Pinoresinol	1.64706	3.62491	3.53953	+	+
42	361.129246	$C_{19}H_{22}O_7$	-0.074	5.6	Ligstroside aglycon	8.21102	14.1568	10.3804	+	+
43	367.361121	$C_{24}H_{48}O_2$	-0.080	3.3	Lignoceric acid	3.1083	3.60292	1.56055	+	-
44	377.124147	$C_{19}H_{22}O_8$	0.056	4.0	Oleuropein aglycon	14.0242	3.16452	13.6205	+	+
45	389.290828	$C_{21}H_{42}O_6$	-0.002	3.0	SFA	9.82473	8.88931	10.6974	+	-
46	391.139885	$C_{20}H_{24}O_8$	0.063	7.8	Methyl oleuropein aglycon	1.88511	4.59536	3.90901	+	+
47	393.119148	$C_{19}H_{22}O_9$	0.324	11.1	Hydroxylated derivative of	2.93417	2.94455	3.05225	+	+

					aldehydic form of oleuropein aglycon					
48	393.155482	$C_{20}H_{26}O_8$	-0.045	3.2	Hydroxylated derivative of ligstroside aglycon derivative	5.61589	9.73995	6.75293	+	+
49	409.15044	$C_{20}H_{26}O_9$	0.100	6.8	Oleuropein aglycon derivative	4.10616	5.18996	3.54232	+	+
50	413.290878	$C_{23}H_{42}O_6$	-0.262	9.5	TG	10.1037	7.23048	10.1697	+	-
51	415.139887	$C_{22}H_{24}O_8$	0.268	14.3	Acetoxypinoresinol	3.94367	6.18115	5.89184	+	+
52	415.306484	$C_{23}H_{44}O_6$	0.017	4.3	MUFA ester	21.0982	13.2781	16.8231	+	-
53	417.155523	$C_{22}H_{26}O_8$	-0.021	10.4	Syringaresinol	1.28993	2.63969	2.32783	+	+
54	417.322155	$C_{23}H_{46}O_6$	-0.005	7.4	SFA ester	6.60908	6.3243	7.14831	+	-
55	423.422612	$C_{28}H_{53}O_2$	0.018-	3.2	Montanic acid	1.50266	1.74077	1.43791	+	-
56	455.353111	$C_{30}H_{48}O_3$	0.045	11.8	Oleanolic acid	2.40333	4.08874	3.93516	+	+
57	471.347941	$C_{30}H_{48}O_4$	-0.263	10.3	Maslinic acid	5.51485	7.47799	6.80188	+	+
58	611.525847	$C_{37}H_{72}O_6$	0.334	64.2	DG derivative	1.2954	2.03549	2.25631	+	-

59	629.491862	$C_{37}H_{70}O_5Cl$	-0.218	31.8	DG	2.65682	2.95243	2.03171	+	-
60	637.541557	$C_{39}H_{74}O_6$	0.383	65.6	Trilaurin	1.08167	1.14146	4.05925	+	-
61	653.542317	$C_{39}H_{73}O_7$	-0.182	54.1	DG derivative	6.05454	5.15802	6.76803	+	-
62	655.515738	$C_{39}H_{72}O_5Cl$	0.304	48.1	DG	3.99641	4.21617	3.60206	+	-
63	679.554723	$C_{41}H_{75}O_7$	-0.07	3.1	DG derivative	7.93679	8.32565	9.20121	+	-
64	849.763319	$C_{53}H_{102}O_7$	-0.114	28.2	TG derivative	1.21126	1.17752	1.31861	+	-
65	867.725262	$C_{53}H_{100}O_6Cl$	0.127	11.3	TG	3.0872	3.7717	1.83018	+	-
66	875.774536	$C_{55}H_{104}O_7$	0.136	17.5	TG derivative	2.87357	2.56699	3.10146	+	-
67	890.714835	$C_{57}H_{110}O_6$	-0.07	2.6	Stearin	1.35101	1.7891	1.03562	+	-
68	891.725417	$C_{55}H_{100}O_6Cl$	0.258	38.9	TG	3.5948	5.35845	3.38906	+	-
69	893.742758	$C_{55}H_{102}O_6Cl$	0.478	33.2	TG	6.5368	2.63229	5.27303	+	-
70	895.732846	$C_{55}H_{104}O_6Cl$	-0.380	52.3	TG	3.65748	4.59915	2.93818	+	-
71	914.753581	$C_{57}H_{98}O_6$	-0.04	1.2	Linolein	1.62813	1.10354	1.40218	+	-
72	919.754312	$C_{57}H_{104}ClO_6$	0.05	5.8	Triolein	6.91628	7.65802	9.66812	+	-

[1]SFA: saturated fatty acid; PUFA: polyunsaturated fatty acid

Table A3: Results of the performed permutation tests. 20 permutations were conducted for each Y-variable.

Type of plot	Y-variable	R2 intercept	Q2 intercept
Figure 2B	Crete	(0.0, 0.441)	(0.0, -0.257)
	Peloponnese	(0.0, 0.4140)	(0.0, -0.257)
	Ionian	(0.0, 0.3900)	(0.0, -0.258)
Figure 2C	Heraklion	(0.0, 0.2677)	(0.0, -0.282)
	Lasithi	(0.0, 0.2400)	(0.0, -0.265)
	Lakonia	(0.0, 0.2130)	(0.0, -0.223)
	Messinia	(0.0, 0.2430)	(0.0, -0.284)
Figure 2D	Crete	(0.0, 0.2650)	(0.0, -0.251)
	Peloponnese	(0.0, 0.2650)	(0.0, -0.272)
	Ionian	(0.0, 0.2820)	(0.0, -0.299)
	Three phases	(0.0, 0.3890)	(0.0, -0.493)
Figure 6A	Conventional	(0.0, 0.6610)	(0.0, -0.333)
	Integrated	(0.0, 0.7010)	(0.0, -0.354)
	Organic	(0.0, 0.6760)	(0.0, -0.366)
Figure 6B	Conventional	(0.0, 0.3610)	(0.0, -0.396)
	Integrated	(0.0, 0.3440)	(0.0, -0.414)
	Organic	(0.0, 0.3920)	(0.0, -0.415)
Figure 8A	Two phases	(0.0, 0.6240)	(0.0, -0.324)
	Three phases	(0.0, 0.7370)	(0.0, -0.353)
Figure 8B	Two phases	(0.0, 0.3730)	(0.0, -0.412)

	Three phases	(0.0, 0.3890)	(0.0, -0.493)
Figure 10	Crete	(0.0, 0.3130)	(0.0, -0.153)
	Peloponnese	(0.0, 0.3250)	(0.0, -0.141)
	Ionian	(0.0, 0.3060)	(0.0, -0.200)

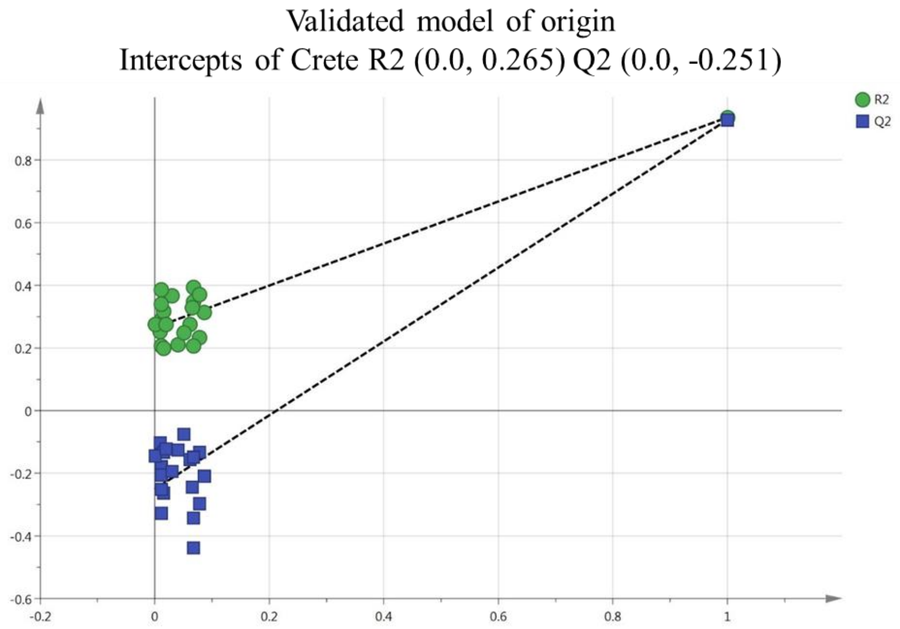

Figure A16: Permutation plot of biophenols OPLS-DA score plot for origin. The plot illustrates the permutation results conducted for figure 4D representing the intercepts of R2 and Q2 parameters for Crete classID.

The permutation plot helps to estimate the validity of the current PLS or PLS-DA. The concept of this validation test is to compare the fitting (R2 and Q2) of the original model with the fitting of several models based on data where the order of Y-observations has been randomly permuted while X-matrix has been kept intact. The above plot shows for a selected Y-variable (origin-Crete) on the vertical axis the values of R2 and Q2 for the original model (far to the right) and of the Y-permutated models further to the left. The horizontal axis shows the correlation between the permuted Y-vectors and the original Y-vector for the selected Y. The original Y has the correlation 1 with itself, defining the high point on the horizontal axis. Figure A8 represents the results from 20 permutation tests conducted for Crete as Y-variable. In the vertical axis the intercepts for R2 (0.0, 0.265) and Q2 (0.0, -0.251) are represneted, indicating the validity and goodness of data fitting of the specific plot.

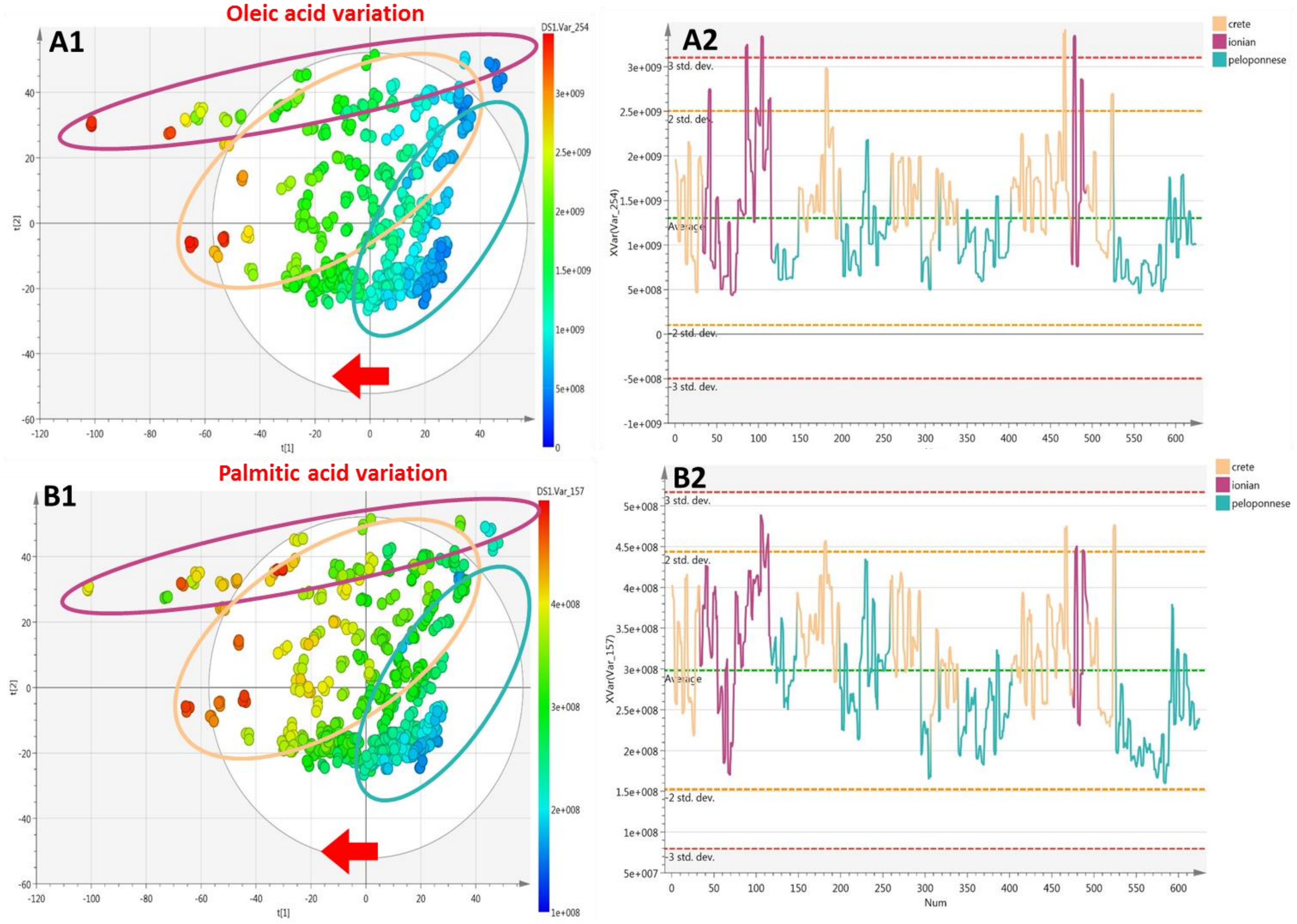

Figure A17: PCA plots of EVOOs colored according to specific X-variables; (A1, B1): PCA plots colored according to the intensity of oleic (A2, var_254) and palmitic acid (B2, Var_157) variable; Diagram illustrating the standard deviations (average ± 3 std. dev.) of oleic (A2, var_257)) and palmitic acid (B2_Var_157) variable. Observations are colored according to the geographical origin; Crete with light orange, Ionian islands with magenta and blue for Peloponnese.

PCA plots in figure represent the variation of oleic and palmitic acid. These metabolites have been derived as statistical significant biomarkers for the parameter of origin. The colored bar (from blue - low intensities to red - high intensities) illustrates the relative intensities for oleic (A1) and palmitic acid (B1) of all observations. Both plots uncover an obvious trend of separation on PC1. Observations on the right are characterized by low relative abundance of the fatty acids (FAs), while observations on the left are characterized by the opposite. Red, yellow and green spots in plot A reflect samples with high and medium intensities of oleic acid and mainly originate from Ionian islands and Crete. In these two areas oleic acid have been identified as statistical significant metabolite. Blue and light blue spots mainly come from Peloponnese peninsula. For plot B the same trend is observed. Based on bibliographic data palmitic acid is formed from oleic acid (1), explaining the same trend. Palmitic acid is found relatively high in Peloponnese. Diagram A2 and B2 is a different visualization illustrating each variable intensity variation in correlation with the calculated standard deviations (average ± 3 std. dev.) in all observations.

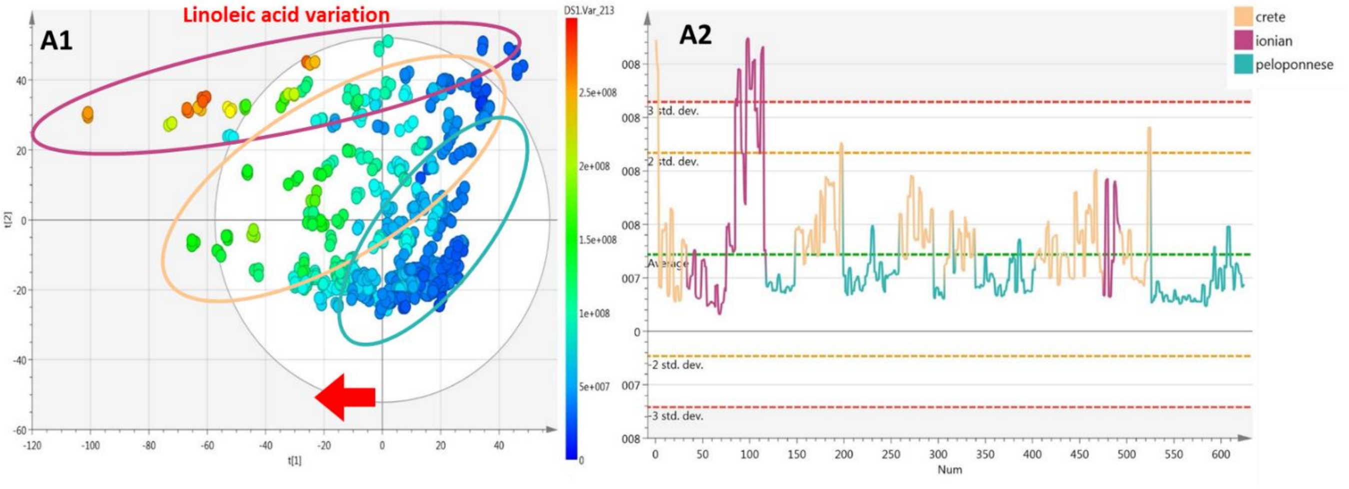

Figure A18: (A1) PCA plot colored according to linoleic acid intensity (pareto scaling); (A2) Diagram illustrating the standard deviation (average ± 3 std. dev.) of linoleic acid variable (var_213). Observations are colored according to the geographical origin; Crete with light orange, Ionian islands with magenta and blue for Peloponnese.

Like palmitic acid, linoleic acid is formed from oleic acid after a desaturation reaction, having an antagonistic relation with palmitic acid formation [1]. In the PCA plot (A1) red and yellow spots are samples originating from Ithaca island and green spots from Cephalonia, meaning that the two Ionian islands of sample collection hold the highest intensities for linoleic acid. On the other hand, Cretan and Peloponnese samples do not contain linoleic acid in considerable amounts, but only in oleic and palmitic acid. Diagram A2 verifies the previous finding, that only in Ionian samples linoleic acid intensities (magenta colored samples) exceed the third standard deviation.

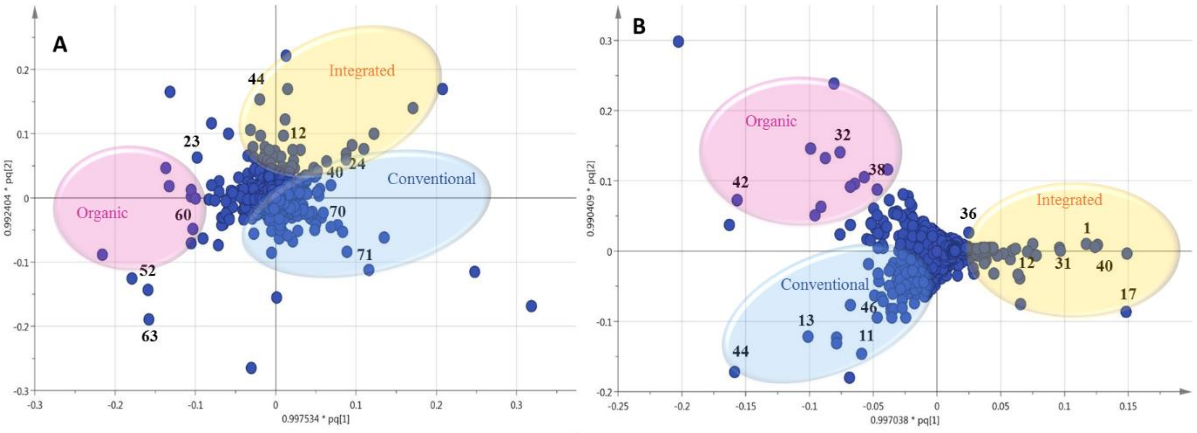

Figure A19: Loadings plots of cultivation practice generated by EVOOs (A) and biophenols (B) dataset. Conventional (blue), Integrated (yellow) and organic (magenta). Representative identified statistical significant metabolites for each practice are annotated.

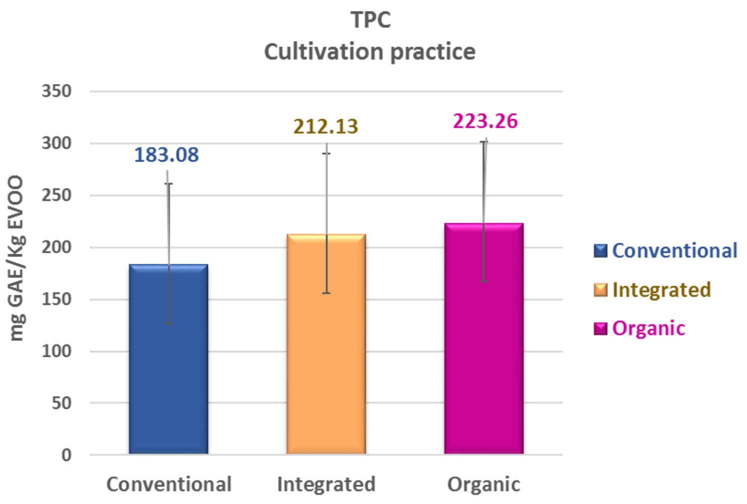

Figure A20: TPC average values versus cultivation practice. Results are expressed in mg GAE/Kg EVOO.

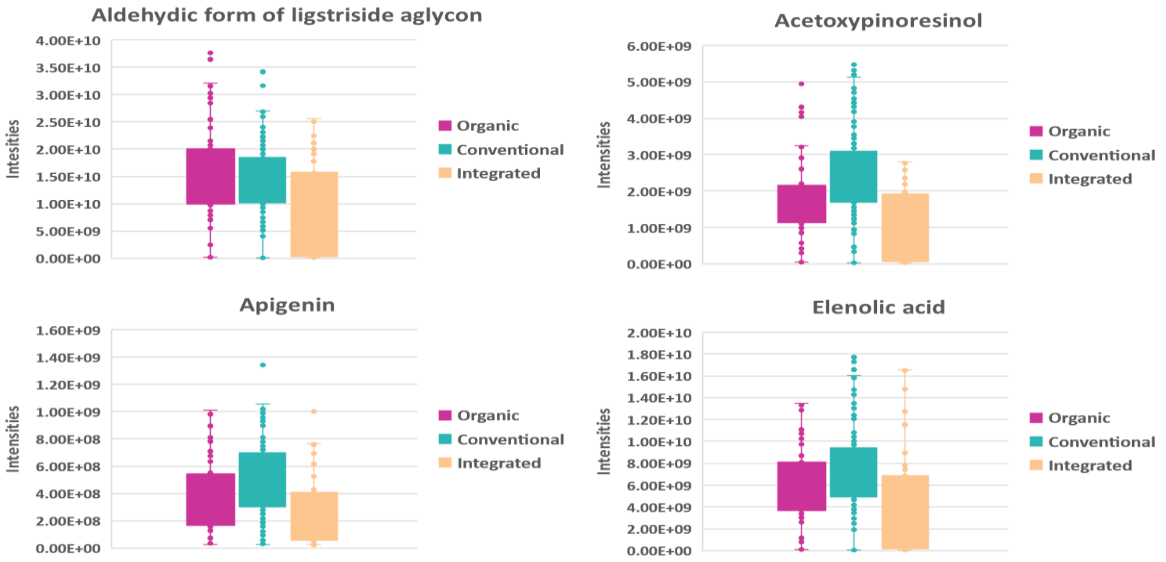

Figure A21: Box-plots representing intensities of individual statistical significant compounds. Box-plots are presented according to cultivation practice; organic (magenta), conventional (blue) and integrated (light yellow).

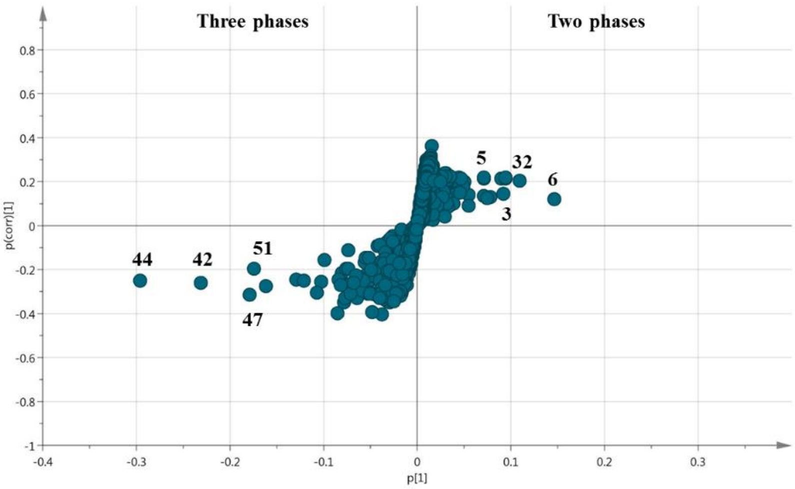

Figure A22: S-plot of production procedure generated by biophenols dataset. In the two treatments statistical significant metabolites are marked.

The S-plot is used for covariance and correlation structure visualization between X-variables and the predictive score t[1]. It is a scatter plot of the p[1] vs p(corr)[1]vectors. This plot often shapes the letter ‘S’ unless the X-variables are scaled to UV. X-variables situated far out the wings of the S combine high model influence with high reliability. In the above S-plot X-variables right to p[1] influence two phases structure visualization, while X-variables left to p[1] influence three phases visualization. In the plot are marked identified compounds revealing high VIP scores for production procedure parameter.

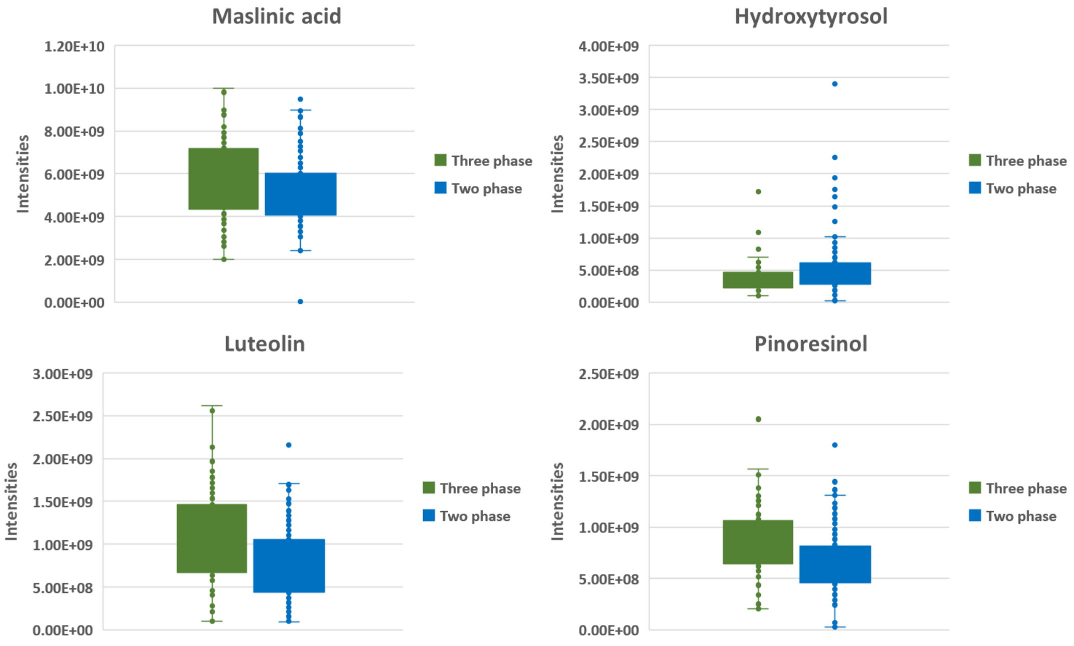

Figure A23: Box-plots representing intensities of individual statistical significant compounds. Box-plots are presented according to production procedure system; three phase (green boxes) and two phase (blue boxes).

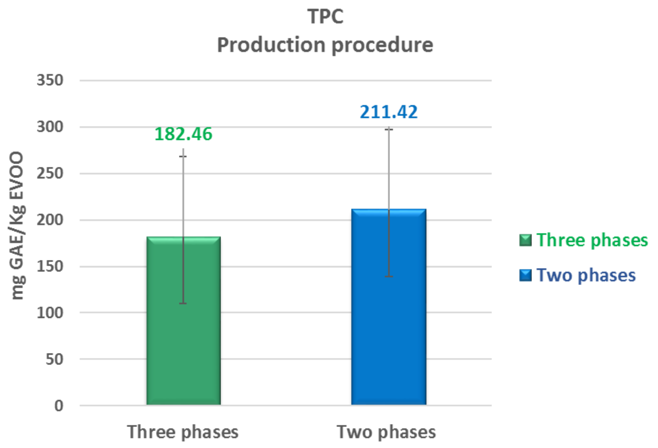

Figure A24: TPC average values versus production procedure system. Results are expressed in mg GAE/Kg EVOO.

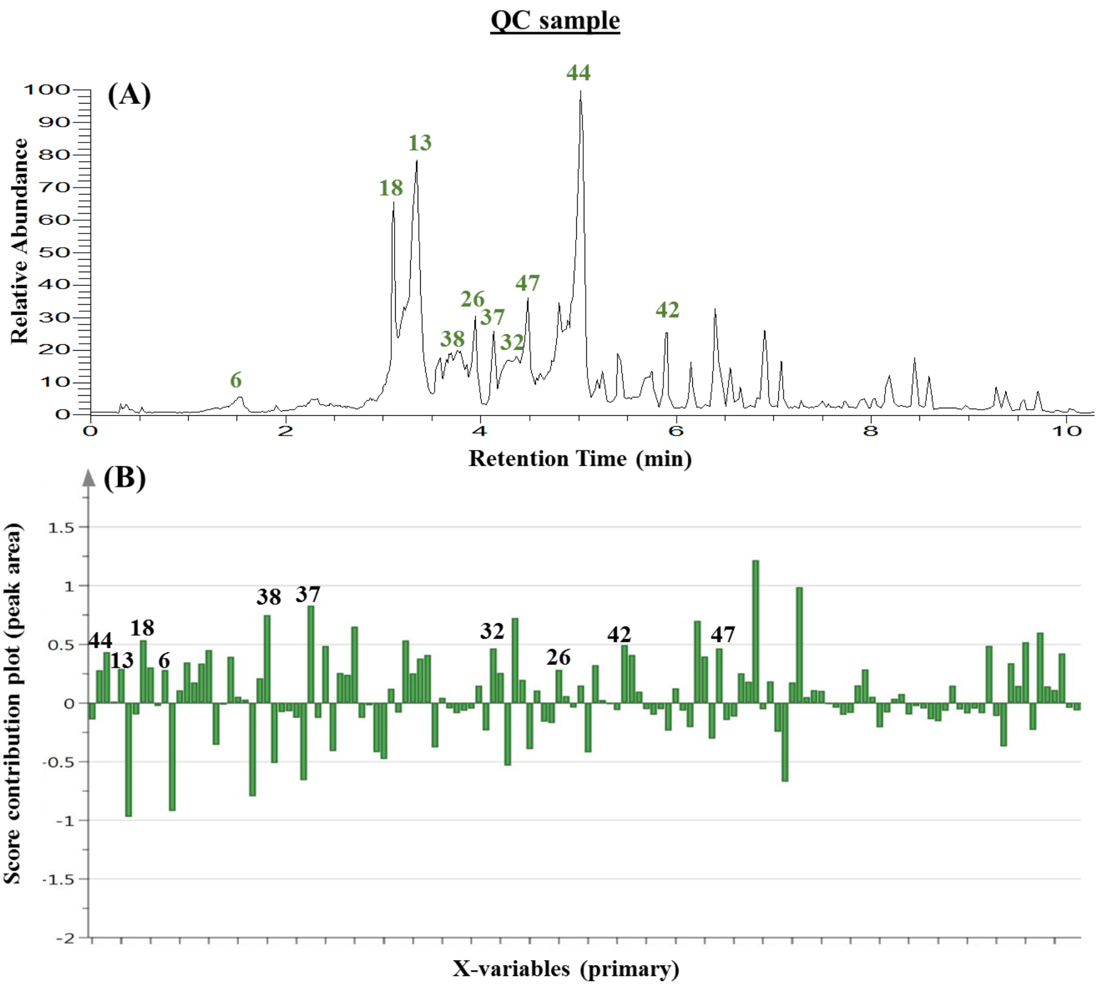

Figure A25: (A)UPLC-HRMS chromatogram of QC sample. (B) Score contribution plot of QC samples. The horizontal scale represents X-variables (*m/z* concatenated to retention time) and the vertical scale is in the units of standard deviations for peak areas of each variable. Representative identified biomarkers are annotated.

Figure A25 associates the peaks of the UPLC-HRMS profile of QC samples with the contribution plot of the generated X-variables (*m/z* concatenated to retention time). Contribution plot (B) interprets the deviation of the variables in QC samples. Their pattern verifying the good fitting and centralization of QCs in the corresponding OPLS-DA plot. The numbers in both plots correspond to compounds with high VIP scores for the discrimination of origin. (presented in detail in Table A2**)**.

Bibliography

(1) V. Ninni, A statistical approach to the biosynthetic route of fatty acids in olive oil : cross-sectional and time series analyses, 2121 (1999) 2113–2121.
